# Tumor immunotherapy and multi-mode therapies mediated by medical imaging of nanoprobes

**DOI:** 10.7150/thno.58413

**Published:** 2021-05-25

**Authors:** Yang Xuan, Meng Guan, Shubiao Zhang

**Affiliations:** Key Lab of Biotechnology and Bioresources Utilization of Ministry of Education, College of Life Science, Dalian Minzu University, Dalian, Liaoning, 116600, China.

**Keywords:** nanoprobes, multi-mode imaging, immunotherapy, combination therapy

## Abstract

Immunotherapy is an effective tumor treatment strategy that has several advantages over conventional methods such as surgery, radiotherapy and chemotherapy. Studies show that multifunctional nanoprobes can achieve multi-mode image-guided multiple tumor treatment modes. The tumor cells killed by chemotherapies or phototherapies release antigens that trigger an immune response and augment the effects of tumor immunotherapy. Thus, combining immunotherapy and multifunctional nanoprobes can achieve early cancer diagnosis and treatment. In this review, we have summarized the current research on the applications of multifunctional nanoprobes in image-guided immunotherapy. In addition, image-guided synergistic chemotherapy/photothermal therapy/photodynamic therapy and immunotherapy have also been discussed. Furthermore, the application potential and clinical prospects of multifunctional nanoprobes in combination with immunotherapy have been assessed.

## Introduction

Cancer incidence and mortality are rapidly increasing world-wide. Most cancers are usually detected at the advanced stages due to lack of effective screening and early diagnosis. In addition, the current therapeutic strategies against cancer have poor outcomes on account of their low efficacy and accuracy, considerable adverse effects, and the high tumor recurrence and metastasis rates. Thus, there is an urgent need to explore new safe and effective techniques of cancer diagnosis and treatment in order to improve patient survival 1.

Currently, malignant tumors are detected through biopsies and imaging. However, a biopsy is invasive, lacks sensitivity and specificity, and cannot be performed routinely due to limited pathological specimens. On the other hand, imaging methods are expensive and cannot detect tumors smaller than 1 cm in diameter 2,3. Therefore, it is crucial to develop advanced imaging techniques for early diagnosis and monitoring of tumors. Nanoprobes, as novel kinds of contrast agents, have been extensively used in medical imaging and drug delivery given their size and optical properties, which translate to high resolution, sensitivity and other advantages 4-8. The nanoprobes with imaging abilities are especially suitable for real-time imaging, which could greatly improve the accuracy of early tumor diagnosis and therapeutic monitoring 9,10. In fact, several nanoprobes have been designed in recent years for the early diagnosis and monitoring of tumors 11.

The aim of cancer treatment is to eliminate the primary tumors, as well as the metastasizing cells to prevent recurrence. However, the complex tumor microenvironment (TME) greatly limits the efficacy of conventional anti-cancer treatment and inhibits complete tumor eradication 12. Cancer immunotherapy is a new generation treatment modality that stimulates the host immune system to selectively kill cancer cells with fewer side effects on healthy tissues, and also promotes systemic immune surveillance that can eliminate both primary and metastatic tumors. Compared to chemotherapy, surgical resection and radiotherapy, the anti-tumor immune cells activated by immunotherapeutic strategies can even kill circulating tumor cells (CTCs) and thus inhibit the formation of metastatic foci. Finally, immunotherapy can establish long-term immune memory to prevent tumor recurrence. Numerous clinical studies have demonstrated that immunotherapeutic agents such as cytokines 13, checkpoint blockers 14, anti-cancer nano-vaccines 15,16 and chimeric antigen receptor T-cells (CAR-T) can effectively treat advanced or metastatic tumors 17. Several tumor immunotherapy drugs have been approved by the Food and Drug Administration of the United States for clinical use as well. However, clinical trials show that single immunotherapy cannot achieve complete tumor remission. For example, the response rate of programmed death receptor-1 (PD-1)/programmed death ligand-1 (PD-L1) checkpoint blockade is only 20%. This is largely attributed to the immunosuppressive TME and the high levels of PD-L1 expressed by the tumor cells, which enable immune escape and survival of the tumor cells at every stage of the immune response 18.

Nevertheless, multifunctional nanoprobes can obviate the above shortcomings 19,20 given their excellent biocompatibility, easy surface modification, and ability to deliver drugs to the target site and protect them against endogenous enzymes 21. Studies show that nanoprobe-assisted radiotherapy, chemotherapy, photothermal therapy (PTT) and photodynamic therapy (PDT) can stimulate the immune system by inducing immunogenic cell death (ICD) 22,23. When combined with immune checkpoint blockade (ICB), the localized therapies can activate tumor-specific immune response to target metastatic cancer cells, and induce immune memory to inhibit tumor recurrence 24,25. Therefore, various therapeutic modalities can significantly augment the effects of immunotherapy against residual tumor cells.

The outcome of cancer treatment is assessed according to the response evaluation criteria in solid tumors (RECIST) by measuring the change in tumor volume and the generation of new tumors 26. However, the infiltration of activated immune cells in the TME can increase tumor volume and delay the response to immunotherapy as per the conventional criteria 27,28. In addition, routine imaging and histopathology cannot accurately predict the clinical response of tumor cells to immune agents 29-31. Multifunctional nanoprobes with magnetic resonance, photoacoustic, fluorescence and other imaging elements can additionally achieve real-time and dynamic visualization of tumors and immune cells, which can not only elucidate the mechanisms of immunotherapy but also accurately monitor and predict patient response 32,33. Furthermore, multi-modal imaging that combine the advantages of several imaging modes can increase the accuracy of tumor diagnosis and monitoring, and clarify the synergistic mechanisms of combination therapy 34.

Thus, multifunctional nanoprobes combining imaging and multimodal treatment can augment immunotherapy and achieve image-guided early diagnosis, precision treatment and real-time monitoring of tumors. In this review, the development and applications of multifunctional nanoprobes have been discussed in detail, with emphasis on image-guided and combination immunotherapy against cancer.

## Image-guided immunotherapy

Multifunctional nanoprobes have unique physical and chemical properties that allow high resolution imaging 35,36. Some inorganic nanoparticles are effective contrast agents for various imaging modes, such as Fe_3_O_4_ nanoparticles for MRI and Au nanoparticles for computed tomography (CT) and photoacoustic imaging (PAI) 37,38. Surface modification of these nanoparticles can additionally endow them with histocompatibility, stability and targeting capacity 39. These nanoparticles can not only be used as contrast agents but also as immune-drug carriers that may overcome the limitations of immunotherapy (Table 1).

**Figure 1:**
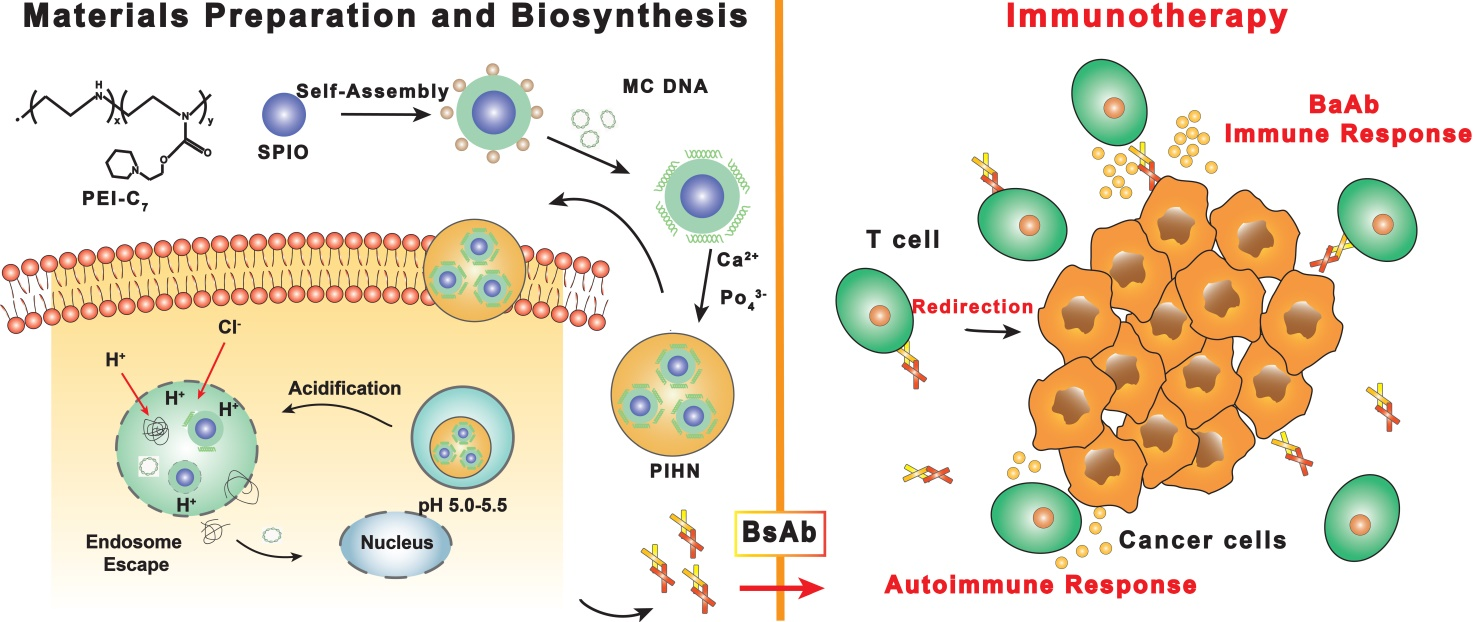
** Schematic illustration of the preparation of polymeric-inorganic hybrid nanocomposites and the bispecific antibody-based immunotherapy.** Adapted with permission from 50, copyright 2019 Elsevier Science Inc. BsAb: bispecific antibody; MC.DNA: minicircle DNA; PIHN: polymeric-inorganic hybrid nanocomposite; PEI: polyethyleneimine; SPIO: superparamagnetic Fe_3_O_4_.

Magnetic resonance imaging (MRI) is a non-invasive technique that uses a magnetic field to obtain nuclear magnetic resonance (NMR) of water protons in the tissue, resulting in three-dimensional images. In a strong magnetic field, hydrogen nuclei absorb resonant radio frequency pulses, and are subsequently excited by the nuclei to relax and return to their original low-energy state 31,49. MRI contrast between tissues is produced by the different relaxation time of spin of the surrounding hydrogen nuclei, which depends on the tissue composition. The MR images of soft tissues are usually high-contrast given the high content of fat and water. Cai *et al.* 50 synthesized PEI-C_7_-SPIO nano-micelles for MRI by coating superparamagnetic Fe_3_O_4_ (SPIO) with pH-sensitive N-(2-hydroxy)-cycloheximine and branched-chain polyethyleneimine (PEI). Minicircle DNA (MC.DNA) was then adsorbed on the nano-micelles through the surface positive charge to generate the polymeric-inorganic hybrid nanocomposite (PIHN) probes (**Figure 1**). The Fe_3_O_4_ nanoparticles functioned as the contrast agents for MRI, whereas the minicircle DNA was transfected into T cells and induced an anti-tumor immune response. The nitrogen/phosphate ratio (N/P) plays an important role in cytotoxicity and transfection efficiency 51. The PIHN probes had low toxicity and resulted in >95% viable cells when the N/P was between 5 and 25. The transfection efficiency of the probes was the highest at the N/P of 25, resulting in maximum bispecific antibody expression. Thus, the PIHN probes have a broad application potential in cancer diagnosis and treatment given the excellent MRI ability of the Fe_3_O_4_ nanoparticles and enhanced T cell activation. In addition to MRI, magnetic particle imaging (MPI) is a more novel imaging technology that has not been used in clinical practice despite its potential. Unlike MRI, MPI requires magnetic nanoparticles (MNPs) to create images with high spatial resolution in the sub-millimeter range, and can therefore detect MNPs with high sensitivity 52.

High-resolution imaging by multifunctional nanoprobes can also elucidate the mechanisms of immunotherapy 53,54 *via* real-time monitoring of immune cells in the TME and the biodistribution of immunomodulatory drugs at the target site 21,32. Rinat *et al.* 55 labeled melanoma-specific T-cell receptor (TCR)-expressing T cells with Au nanoparticles (AuNNPs) for CT image-guided therapy. AuNNPs loading did not affect the T cells proliferation and cytokine secretion *in vitro*. Furthermore, the migration and accumulation of the AuNNPs-loaded T cells at the tumor site was clearly observed by high-resolution CT. The functionalized T cells significantly reduced the tumor volume in the melanoma-bearing nude mice within 7 days post-injection, indicating excellent tumor-inhibitory effect. This study was the first to use real-time CT imaging to track labeled immune cells, and assess their biodistribution, migration and eventual fate, which provides new insights into the interaction between immune cells and tumor cells 55.

ICB is the most widely applied anti-tumor immunotherapeutic strategy. PD-L1 is an immunosuppressive molecule that inhibits T cell activation upon binding to PD-1 56, and the PD-1/PD-L1 pathway is the major mechanism of immune tolerance. PD-L1 is a therapeutic target in breast cancer given that almost 20% of the patients express PD-L1 57,58 and its high expression levels correlate with poor prognosis. Monoclonal antibodies (mAb) targeting PD-L1 have been approved by the US Food and Drug Administration (FDA) for the treatment of various cancers 59,60. Several groups have imaged T cells and lymphoid tissues in mice using radioisotope-labeled PD-L1 and PD-1 antibodies 61,62. However, despite the high affinity, strong antigen specificity and easy synthesis of radio-labeled antibodies, the prolonged circulation, high background signal and poor tumor penetration obviate imaging within 24 hours of tracer injection. On the other hand, fluorescence imaging (FI) has the advantages of rapid detection, high sensitivity and non-invasiveness. Samit *et al.* 63 coupled the near infrared (NIR) fluorescent dye Licor800 to PD-L1-mAb to detect the expression of PD-L1 on different breast cancer cell lines, and recorded strong fluorescence intensity for the PD-L1-positive MDA-MB-231 cells as opposed to the weak fluorescence for the PD-L1-negative SUM149 cells. Thus, NIR-PD-L1-mAb can detect PD-L1-expressing tumor cells with high specificity, and is a promising tool for the optical imaging of these tumors.

**Figure 2:**
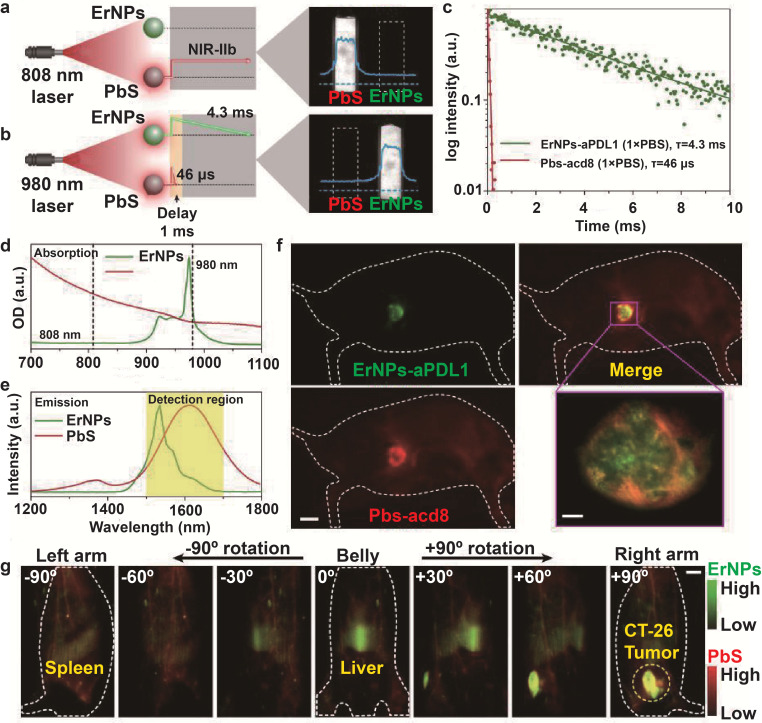
***In vivo* two-plex NIR-IIb molecular imaging of immune responses using ErNPs-aPDL1 and PbS-aCD8 at the same ~1,600 nm emission range. A.** Schematic illustration outlining the experimental setup (left) to distinguish the PbS QD emission channel (right) by using an 808 nm CW laser. **B.** Schematic of the experimental setup (left) to differentiate the long-lived ErNP luminescence (right) from short-lived PbS QD fluorescence by using a 980 nm laser pulse. The insets show corresponding cross-sectional intensity profiles (blue color). **C.** Lifetime decays of ErNPs-aPDL1 and Pbs-aCD8 in 1 × PBS solution. **D.** Absorption spectra of ErNPs-aPDL1 and PbS-aCD8. **E.** Emission spectra of ErNPs-aPDL1 and PbS-aCD8. The detection region is 1,500 - 1,700 nm. **F.** Two-plex molecular imaging (upper right) of a CT-26 tumor mouse at 24 h post intravenous injection of mixed ErNPs-aPDL1 (green color, upper left) and PbS-aCD8 (red color, lower left). Scale bar, 5 mm. The zoomed-in high-magnification two-plex image (lower right) outlines the CT-26 tumor with micrometer image resolution (scale bar, 500 μm). **G.** Corresponding two-plex rotation (-90º to +90º) imaging showing the *in vivo* bio-distribution of ErNPs-aPDL1 (green color) and PbS-aCD8 (red color) in the whole body. Scale bar, 5 mm. Similar results for n > 3 independent experiments. OD, optical density. Adapted with permission from 43, copyright 2019 Nature Publishing Group. ErNPs: cubic-phase (α-phase) erbium-based rare-earth nanoparticles; ErNPs-aPDL1: anti-PD-L1 mAb (atezolizumab) conjugated with ErNPS; PbS QDs: sulfide quantum dots; Pbs-aCD8: anti-CD8α mAb (clone 2.43) conjugated with Pbs QDs.

In recent years, the second near infrared (NIR-II, 1000-1700 nm) window has been used more frequently for *in vivo* imaging. Compared to routine FI that operates within the wavelength range of 400-900 nm, the longer wavelength of NIR-II reduces photon absorption and scattering, resulting in minimal tissue autofluorescence. In addition, NIR-II FI has deeper tissue penetration, and higher spatial and temporal resolution 64,65, which makes it ideal for deep tissue imaging 43,66. Yeteng *et al.* 43 developed biocompatible cubic-phase (α-phase) erbium-based rare-earth nanoparticles (ErNPs) exhibiting bright down-conversion luminescence at ~1600 nm for dynamic imaging of cancer immunotherapy. The ErNPs functionalized with anti-PD-L1 antibodies imaged PD-L1 colon tumors in a mouse model with tumor to normal tissue signal ratio of almost 40. Since the outcome of ICB depends on the activation of tumor-specific cytotoxic T lymphocytes (CTLs) that infiltrate the tumor tissues and induce apoptosis of cancer cells 67,68, the PD-L1-targeting ErNPs were administered along with anti-CD8 α-mAb-labeled lead sulfide quantum dots (PbS QDs) to simultaneously locate CD8^+^ CTLs and tumor cells by luminescence at 1600 nm. The combination of the long-life luminescence (4.3 ms) of ErNPs and short-life luminescence (46 μs) of PbS QDs at the same emission wavelength further improved temporal resolution (**Figure 2**), and showed the presence of CD8^+^ CTLs in the TME and spleen, clearly indicating immune activation. At present, the proportion of PD-L1-expressing tumor cells and tumor infiltrating lymphocytes (TILs) after immunotherapy are detected by biopsies to predict treatment response. However, biopsies are invasive and often difficult to obtain from deeper tumor tissues, which can result in misdiagnosis. Bimolecular imaging by ErNPs and PbS QDs is a non-invasive alternative that can accurately track distribution of PD-L1 tumor cells and CTLs.

**Figure 3:**
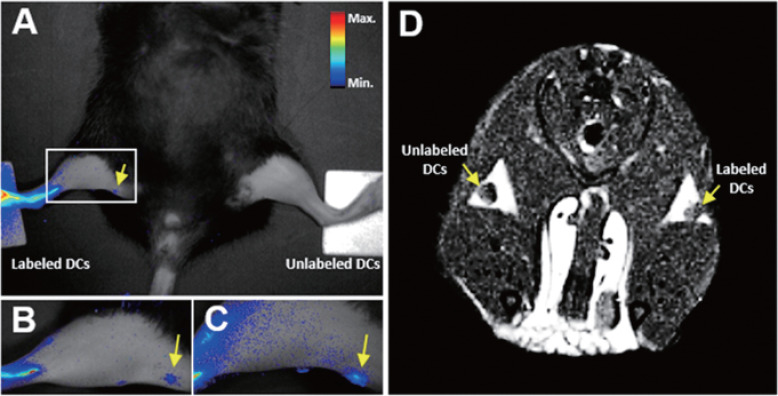
***In vivo* NIR and MRI tracking of the migration of AB-BCA-treated BMDCs to LNs in mice.** BMDCs treated with or without AB-BCA were subcutaneously injected in both hind footpads at a density of 5 × 10^6^ cells/mouse. **A.** NIR fluorescence images of the whole body at 24 h after injection. **B.** NIR fluorescence images of the magnified thigh at 24 h after injection. **C.** NIR fluorescence images of the magnified thigh at 48 h after injection. **D.** MR images at 24 h after injection. Arrows indicated the LNs. Adapted with permission from 45, copyright 2016 Amer Chemical Soc.

Single-mode imaging of tumors is usually ineffective due to one or more limitations. For example, MRI has high spatial resolution but limited sensitivity, whereas FI has excellent sensitivity but lacks spatial and anatomical resolution 69,70. Dual-mode imaging can significantly improve the efficiency of tumor diagnosis by compensating for the limitations of each mode 71,72. Several multi-mode imaging methods have been developed in recent years to improve the accuracy of early cancer diagnosis 44,73. Du *et al.* 44 developed the theranostic PD-L1-PCI-Gd nanoprobes that combined MRI/FI dual-mode imaging with PD-L1 targeting. The fluorescence intensity of PD-L1-targeted nanoparticles increased steadily in 4T1 tumors, and the final fluorescence intensity was almost 2-fold higher than that of the background. Similarly, MRI of 4T1 tumors showed higher signal intensity than that of the control group. The multifunctional dual-imaging nanoprobes can improve the accuracy of early tumor diagnosis with high sensitivity and spatial resolution. In addition, non-invasive imaging can also be used to monitor cell-based therapy and evaluate the immune microenvironment, which is critical to improve the efficacy of immunotherapy. Eun *et al.* 45 developed the MRI/NIR FI dual-imaging aza-BODIPY-based contrast agent (AB-BCA) nanoprobe by combining Gd-chelating MR probe and NIR fluorophore aza-BODIPY, which was able to trace macrophages and dendritic cells (DCs) *in vivo* (**Figure 3**). The probes were easily internalized by the phagocytic cells, and allowed continuous monitoring of the labeled cells through strong fluorescence and MRI signals without any obvious cytotoxicity.

Natural killer (NK) cells are innate immune cells that form the early defense barrier against pathogens and tumor cells 74,75. Since exogenous substances are not easily internalized by non-phagocytic cells, it is difficult to label NK cells with imaging probes. Yong *et al.* 46 combined the MRI agent perfluorodecalin (PFD) with fluorescent InGaP/ZnS QDs to form PFD/[InGaP/ZnS QDs] dual-imaging nano-emulsions that were successfully internalized into immune cells such as macrophages, DCs and NK cells without any transfection reagent. The nanoprobes had almost no effect on cell viability and function, indicating potential applications in image-guided immunotherapy.

Tumor heterogeneity is a hallmark of cancer, and a major factor limiting therapeutic efficacy. Tumor cells with distinct genotypes co-exist in the same tumor, and significantly affect patient prognosis and treatment response. Therefore, different cancer patients respond inconsistently to the same treatment regimen 76,77, and monotherapies such as surgery, chemotherapy, radiotherapy, immunotherapy, PTT and PDT cannot achieve complete tumor cessation. Therefore, combination therapies (**Table 2**) that can reduce some adverse effects of particular monotherapies and result in stronger anti-tumor effects through the complementary action of different treatment mechanisms are increasingly gaining precedence 78,79. Studies show that chemotherapy with doxorubicin (DOX) or oxaliplatin (OXA) can trigger an immune response *via* ICD of tumor cells 22,80, which increases the surface expression of pro-apoptotic calreticulin (CRT), promotes the maturation of antigen-presenting cells (APCs) and increases accumulation of CTLs in the tumors 81,82. In addition, PDT and PTT can also stimulate the immune system by inducing ICD 83,84. The combination of these monotherapies with ICB can target the metastatic cancer cells and residual tumor cells, and promote expansion of memory immune cells to inhibit tumor recurrence. Therefore, chemotherapy, PDT or PTT combined with an immunoadjuvant can significantly augment the effects of immunotherapy 82.

## Image-guided chemo-immunotherapy

Chemotherapy is not only limited by low tumor specificity and adverse effects, but also diminishes the anti-tumor immune response by inhibiting production of white blood cells in the bone marrow 85. However, targeted chemotherapy can enhance the anti-tumor immune response by promoting the activation of tumor-associated DCs and the expansion of effector T cells, and augment the effects of immunotherapy 86,87. In addition, multifunctional nanoparticles with chemotherapeutic drugs and imaging probes allows tumor-targeted drug delivery, real time monitoring of the nanoprobes and non-invasive controlled drug release at the target site 40,80. Another advantage of image-guided, stimuli-responsive drug release is that the time window of treatment can be optimized 89,90.

**Figure 4:**
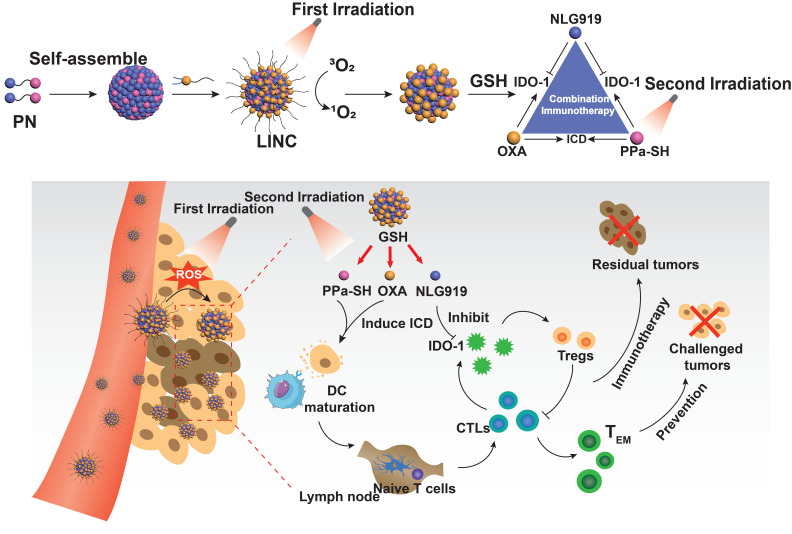
** Schematic illustration of NIR light-inducible LINC for self-amplified drug delivery and combination immunotherapy.** Arrows indicated the LNs. Adapted with permission from 82, copyright 2019 Wiley-V C H Verlag Gmbh. CTLs: cytotoxic T lymphocytes; IDO-1: indoleamine 2-dioxygenase 3-dioxygenase 1; ICD: immunogenic cell death; LINC: light-induced immunotherapy nano-drug; OXA: oxaliplatin; PN: indoleamine 2-dioxygenase 3-dioxygenase 1 (IDO-1) inhibitor NLG919; GHS: glutathione; PPa: photosensitizer pheophorbide A.

Bing *et al.* 82 synthesized a light-induced immunotherapy nano-drug (LINC) consisting of photosensitizer pheophorbide A (PPa), redox-responsive heterodimer of indoleamine 2-dioxygenase 3-dioxygenase 1 (IDO-1) inhibitor NLG919 (PN), and light-activatable prodrug oxaliplatin (OXA) (**Figure 4**). LINC accumulated at the tumor site and emitted NIR fluorescence signals following intravenous injection in mice. Furthermore, the first FI-guided NIR laser irradiation induced the production of reactive oxygen species (ROS) and cleavage of polyethylene glycol (PEG) canopy, which significantly increased the retention and penetration of LINC into the deeper tumor layers, resulting in 2.5-fold stronger fluorescence intensity compared to the non-irradiated group 24 h post-injection. After the second NIR laser irradiation, LINC promoted anti-tumor immunogenicity by promoting infiltration of CTLs in the tumor. In addition, NLG919 reversed the immunosuppressive TME by activating IDO-1. Taken together, LINC inhibited tumor growth and lung metastasis by chemo-immunotherapy and induced continuous immune memory to prevent tumor recurrence. The targeted accumulation and retention of LINC was monitored by *in vivo* FI and PAI. The latter also showed that LINC was mainly distributed in the peripheral regions of the tumor in the non-irradiated mice, whereas laser irradiation increased LINC diffusion into the deeper tumor region within 2 h of injection.

Despite the aforementioned advantages of nanoplatforms, the pathophysiological barriers of solid tumors limit the accumulation of drug-loaded nanoparticles, which in turn lowers therapeutic efficacy. Multifunctional nanoprobes that can release the chemotherapeutic drug in a sustained manner at the target site can significantly improve the treatment efficiency and reduce damage to normal tissues 91. Jin *et al.* 92 synthesized melittin-RADA 32 hybrid polypeptide hydrogel and loaded it with the antineoplastic drug DOX. The melittin-RADA32-DOX (MRD) nanogel was further functionalized with curcumin, paclitaxel and optical contrast agents such as Cy7 and indocyanine green, with a loading rate close to 100%. The hydrogel showed excellent biocompatibility and controlled release of mellitin and DOX, which inhibited melanoma growth in a mouse model by 95% after a single injection with minimal toxicity. The antigens released by the dying tumor cells were engulfed and presented by DCs in the draining lymph nodes, resulting in the activation of specific CTLs that killed the residual tumor cells. The distribution and breakdown of the hydrogels were monitored by the NIR fluorescent dye Cy7 (Cyanine7), which has a molecular weight similar to that of DOX. Xia *et al.* 93 synthesized multifunctional FI nanoprobes by loading DOX into CpG self-crosslinked hydrogel nanoparticles. Co-stimulation by DOX and the immunogen CpG reversed the immunosuppressive TME and induced a stronger immune response, thus achieving efficient chemo-immunotherapy. In addition, compared to the direct hydrogel-mediated delivery of DOX, the CpG self-crosslinked hydrogel nanoparticles achieved more sustained immunostimulatory effect. FI confirmed the targeted delivery of the nanohydrogel, which significantly reduced any adverse effects of the loaded drugs.

**Figure 5:**
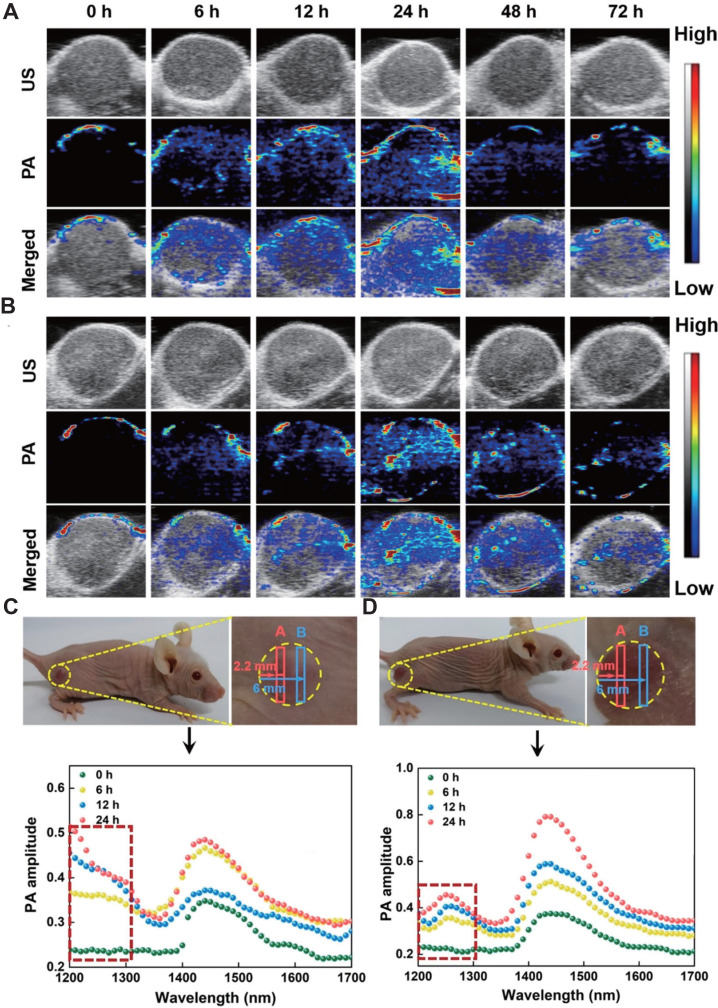
***In vivo* PAI-guided monitoring of BLZ-945 and drug release. A.** PA images of MCF-7 tumor-bearing Balb/c nude mice treated with AuNNP@SN38/BLZ-945 Ve at different time points post-injection. **B.** PA images of MCF-7 tumor-bearing Balb/c nude mice treated with AuNNP Ve at different time points post-injection. **C.** The PA spectra of the tumors treated with AuNNP@SN38/BLZ-945 Ve at different time points in the photograph of tumor bearing mice. **D.** The PA spectra of the tumors treated with AuNNP Ve at different time points in the photograph of tumor bearing mice. Adapted with permission from 90, copyright 2020 Amer Chemical Soc.

Blocking monocyte recruitment and the subsequent activation of tumor-associated macrophages (TAMs) *via* inhibition of CSF-1/CSF-1R is a novel immunotherapeutic strategy 94,95. The small molecule BLZ-945 specifically inhibits CSF-1R phosphorylation and blocks CSF-1-mediated signal transduction in TAMs, leading to apoptosis and improving CTC infiltration in the tumor 96,97. However, free BLZ-945 has a poor tumor inhibition effect, which necessitates dual-responsive nano-delivery systems to drug delivery 98-100. Rong *et al.* 90 loaded BLZ-945 into self-assembled AuNNP@PEG/PSN38VP probes synthesized by grafting AuNNPs with polyethylene glycol (PEG) and pH/GSH (glutathione)-responsive 7-ethyl-10-hydroxycamptothecin (SN38). The AuNNP@SN38/BLZ-945Ve particles dissociated into the hydrophilic AuNNP@PEG/PSN38VP in the acidic TME and released BLZ-945. The smaller AuNNP@PE-G/PSN38VP penetrated into the deeper tumor regions and released the SN38 prodrug in the reductive environment, leading to tumor cell apoptosis. The strong plasmonic coupling between AuNNP significantly enhanced the localized surface plasmon resonance (LSPR) absorbance in the NIR-II window, and the resulting PA signals enabled high contrast PAI for monitoring drug release and treatment (**Figure 5**). The multifunctional nanoprobes achieved PAI-guided simultaneous chemo-immunotherapy to inhibit the growth of primary and metastatic tumors, and prolonged the survival of tumor-bearing mice. Thus, AuNNP@SN38/BLZ-945Ve is an excellent dual-responsive nanoplatform with broad application prospects in drug delivery and bioimaging.

## Image-guided PTT and immunotherapy

Compared to conventional anti-cancer treatments, PTT has certain advantages like high specificity, minimal invasiveness and precise spatiotemporal selectivity 101. Several nanoparticles have been developed in recent years that integrate contrast agents and photoabsorbers into a single platform for simultaneous diagnosis and PTT 102,103. Image-guided PTT can effectively ablate primary tumors with little damage to surrounding tissues, but has a poor therapeutic effect on metastatic tumors. Nevertheless, the antigens released from photothermally ablated tumor cells can trigger systemic anti-tumor immunity. The combination of PTT and immunotherapy can synergize their therapeutic effects on distant primary tumors and metastatic cancer cells, and enhance anti-cancer immunity with less invasiveness and shorter course 101. Local hyperthermia also trigger the release of immunomodulatory molecules 29,104, upregulate MHC-I, MHC-II, CD80 and CD86 on DCs, and increase their migration to draining lymph nodes, thereby enhancing the ability of DCs to present antigens to T cells. In addition, local hyperthermia increases blood flow and vascular permeability to promote infiltration of T cells into the tumor, which further augments the anti-tumor immune response and delays tumor growth 105. However, lymphocyte infiltration into the deeper layers of solid tumors is inadequate due to several immune escape mechanisms, which can only be obviated by reversing immunosuppression and inducing stronger ICD.

Yinchu *et al.* 106 achieved more uniform and deeper ICD in solid tumors with PTT using NIR-II, resulting in stronger innate and adaptive immune responses to control tumor growth and prevent metastasis. Photothermal conversion was mediated by Au nanoparticles self-assembled on fluid liposomes, and exhibited similar composition, structure and conversion efficiency, but differed in terms of absorption in the red light, NIR-I and NIR-II window. One study showed that PTT using wavelengths of all regions induced ICD and released damage associated molecular patterns (DAMPs) *in vitro* 107, although only NIR-II PTT released DAMPs from the deeper tumor regions *in vivo* and completely inhibited tumor growth in most mice by triggering both innate and adaptive immune responses.

**Figure 6:**
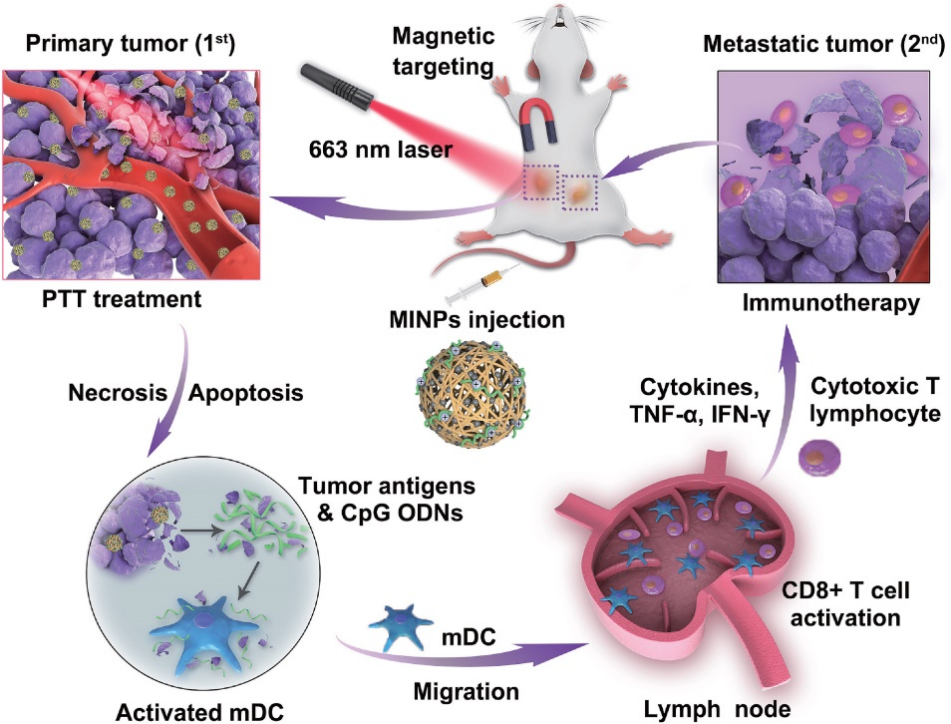
** Schematic illustration of image-guided photothermally triggered immunotherapy using magnetic-responsive immunostimulatory nanoagents (MINPs) for both primary treated and distant untreated tumors.** Adapted with permission from 112, copyright 2019 Elsevier Sci Ltd. CpG ODNs: cytosine-phosphate-guanine oligodeoxynucleotides; IFN-γ: interferon gamma; MINPs: magneto-responsive multifunctional nanoprobes; PTT: photothermal therapy; TNF-α: tumor necrosis factor-α.

Adjuvants are drugs or bioactive substances that enhance immune response to antigens, and have also been tested in tumor immunotherapy 108,109. Hydrogels are highly suitable drug carriers given their excellent good biocompatibility and slow drug release 110. Cytosine-phosphate-guanine (CpG) oligodeoxynucleotides are potent immunostimulants that have proved to be effective as adjuvants in tumor immunotherapy 111. Dong *et al.* 108 synthesized CpG nanoparticles (CpG NPs) by crosslinking CpG and PEI in the presence of genipin and conjugated them with indocyanine green (IR820)-loaded hydrogels for combined PTT and immunotherapy against melanoma. IR820 induced tumor antigen release *via* the photothermal effect, and CpG augmented the ensuing anti-tumor immune response. In addition, the distinct fluorescence signals of genipin and IR820 hydrogels could be individually monitored *in vivo* for 12 days, which revealed that the photothermal action of IR820 hydrogel not only accelerated the degradation of CpG NP_S_ but also enhanced their persistence to achieve an effective immune response. Yuan *et al.* 112 designed magneto-responsive multifunctional nanoprobes (MINPs) loaded with CpG and SPIO for PA/MR dual-mode imaging-guided PTT and immunotherapy (**Figure 6**). MINPs functioned as MRI contrast agents and enabled targeted accumulation of SPIO and CpG in the tumors under an external magnetic field, thus achieving accurate dual-mode imaging. The photothermal effect of MINPs effectively destroyed the primary tumor and released tumor-associated antigens, thereby acting as an “autologous tumor vaccine”. The adjuvant action of CpG further enhanced the effect of immunotherapy to achieve a more effective systemic treatment effect than PTT or immunotherapy alone.

**Figure 7:**
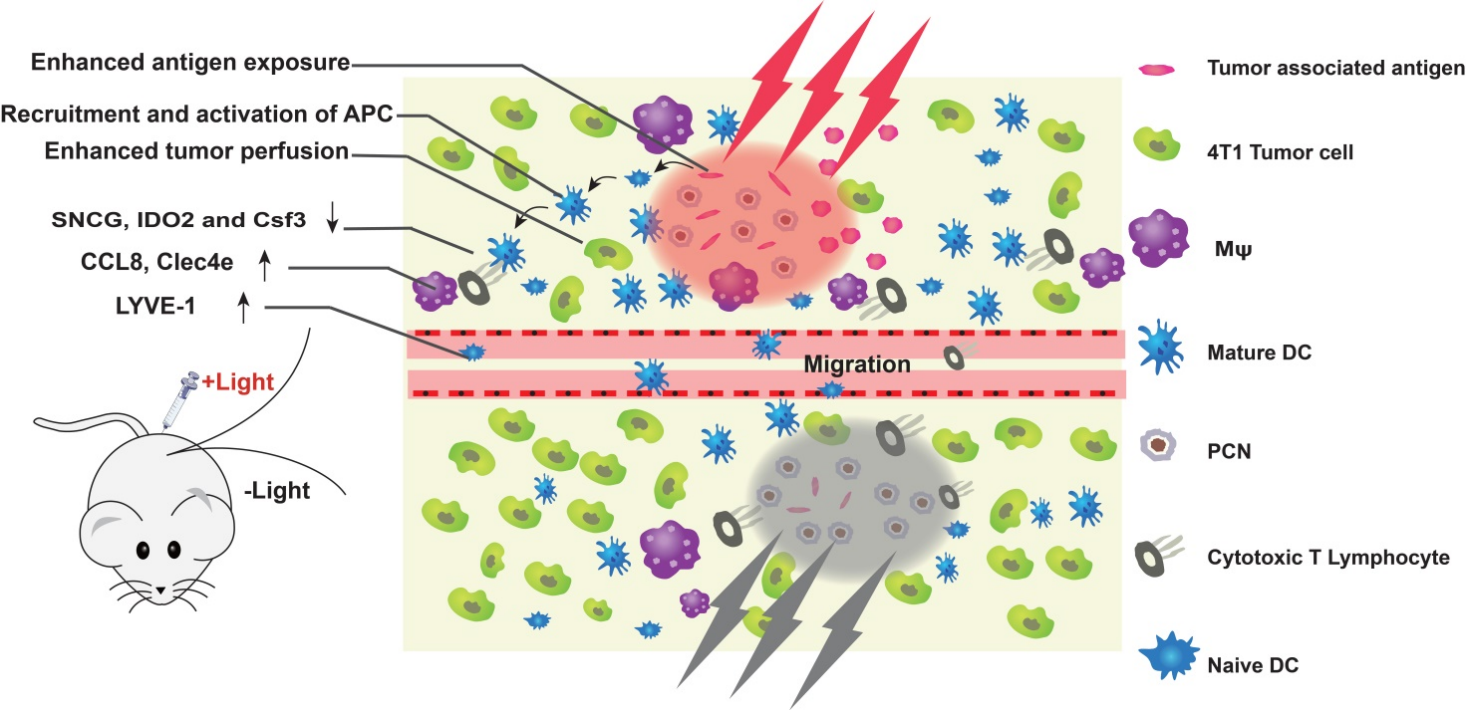
**An immune-favorable TME established *via* fever-like immune-response induced by the photothermal effect of PCN.** Adapted with permission from 113, copyright 2018 Wiley. APC: antigen presenting cells; Csf3: colony stimulating factor 3; CCL8: c-c motif chemokine ligand 8; Clec4e: c-type lectin domain family 4 member e; DC: dendritic cell; IDO2: indoleamine 2,3-dioxygenase 2; LYVE-1: lymphatic vessel endothelial hyaluronan receptor 1; Mφ: macrophages; PCN: photothermal CpG nanotherapy; SNCG: γ-synuclein.

The efficacy of ICB-based cancer immunotherapy depends primarily on the expression of PD-L1 in tumor tissues and the recruitment of TILs. The immunologically “cold” tumors with poor lymphocyte infiltration do not respond to immune checkpoint inhibitors 113. Although mild PTT (about 45 °C) can function as an adjuvant in immunotherapy, it can also up-regulate heat shock proteins (HSPs), indoleamine 2,3-dioxygenase (IDO) and PD-L1 on the tumor cells, which aid in immune escape. Liping *et al.* 114 proposed a combination of mild PTT and anti-PD-L1 antibody to reprogram the “cold” TME and sensitize the tumors to ICB, thereby achieving photothermal immunotherapy. Yan *et al.* 113 developed photothermal CpG nanotherapy (PCN) to modulate the TME using mild heat (**Figure 7**). Ovalbumin (OVA) was assembled with Au nanorods through free mercaptan and disulfide bonds, and CpG was selectively integrated through interaction with OVA and coordination effect of terminal thiol group with Au nanorod. The multifunctional nanoprobes exhibited good biocompatibility and biodispersion, and significantly increased apoptosis and necrosis of tumor cells under light stimulation. The latter released tumor antigens, which promoted anti-tumor immunotherapy in coordination with CpG. Local mild hyperthermia further augmented the immune response, thereby promoting the activation/maturation of innate immune cells. Numerous CD8^+^ T lymphocytes and high IL-6 levels were detected in the light-treated PCN group. In addition, mild hyperthermia down-regulated the expression of several immunosuppressive genes (SNCG, IDO2 and Csf3) in the CD8^+^ T cells, which significantly improved the infiltration, proliferation and activation of CTLs. These results suggest that local PTT-induced immune responses are usually weak and limited by the immunosuppressive TME, which can be overcome by including an immune adjuvant. Thus, a combination of mild PTT and immunotherapy can synergistically eliminate primary tumors, suppress metastasis and prevent tumor recurrence 25,115.

However, most lasers can only target the superficial tumors, which limits the hyperthermic effect in the deeper tumor tissues. Magnetic hyperthermia (MHT) of solid tumors can be achieved through a remote-controlled alternating magnetic field (AMF), which can penetrate deeper tissues more safely and efficiently compared to light and other sources of heat. Magnetic iron oxide nanoparticles (MIONs) repeatedly rearrange the magnetic moments of the ions under AMF, likely due to the internal spin shifting from one direction to another (Néel relaxation) or the physical rotation of the particles (Brownian relaxation). In both cases, this reorientation leads to the conversion of electromagnetic energy into thermal energy, making MIONs controllable heat sources 101. The heat generated by the interaction of MIONs with magnetic fields induces apoptosis or necrosis in the tumors, which in turn stimulate an immune response 116. Maintaining T cell activity after activating the immune response is also the key to obtaining antitumor immunity. Combining checkpoint inhibitors with hyperthermia to avoid immune escape has been confirmed to provide significantly improved antitumor immunity 117. MHT can overcome poor penetration of PTT into deeper tumor tissues, and should be explored as an alternative for combined PTT and immunotherapy.

## Image-guided photodynamic and immunotherapy

PDT is a non-invasive tumor ablation method that relies on cytotoxic ROS production by photosensitizers (PSs), eventually leading to the apoptosis and necrosis of tumor cells 118. However, traditional PSs have poor targeting ability and water solubility, and are easily destabilized *in vivo*. In addition, PDT is also ineffective against the deep-seated and metastatic tumors due to limited tissue penetration of the laser sources and the hypoxic microenvironment, thus precluding its widespread application. Nano-PDT platforms that deliver PSs using targeted nanoprobes can not only improve the stability, biocompatibility and accessibility of PSs, but also increase therapeutic efficacy and reduce side effects 119. On the other hand, PDT-induced tumor cell death releases DAMPs that include multiple tumor-specific antigens, which are then presented by the DCs and other APCs to the CD4^+^ and CD8^+^ T cells 120,121, thereby eliciting an anti-tumor immune response. Image-guided PDT can improve its therapeutic efficacy by elucidating the tumor size and location, and the optimal treatment time 122.

**Figure 8:**
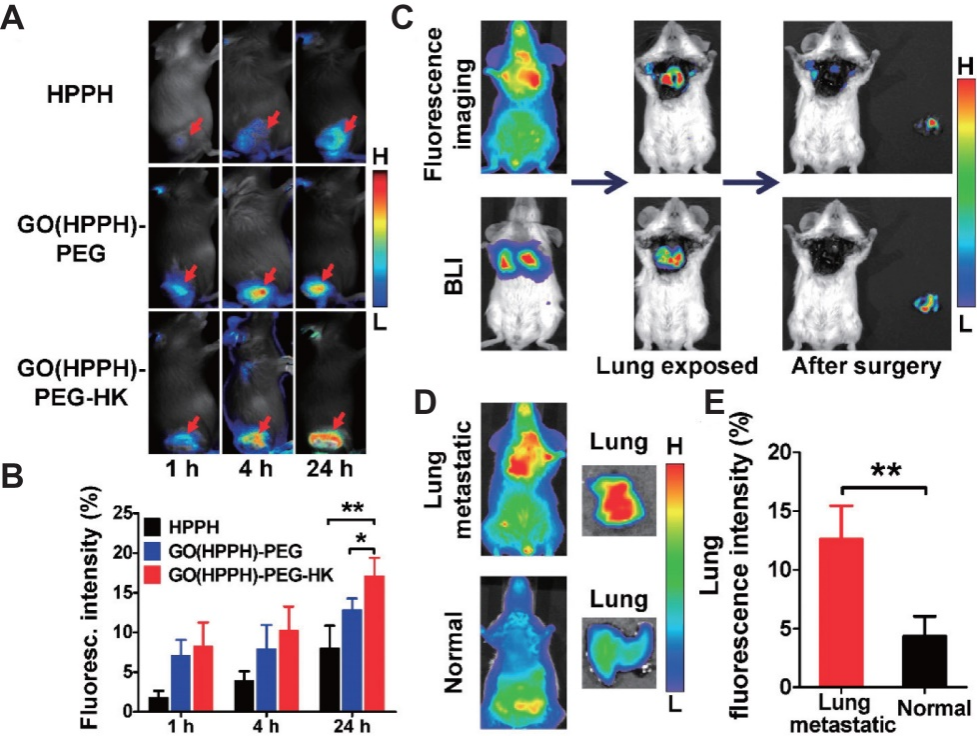
**A.** Representative optical images of 4T1 tumor-bearing BALB/c mice at 1, 4, and 24 h after injection of HPPH, GO(HPPH)-PEG, or GO(HPPH)-PEG-HK. Tumors are indicated by arrows. **B.** Quantitative analysis of HPPH, GO(HPPH)-PEG, and GO(HPPH)-PEG-HK uptake by 4T1 tumors at 1, 4, and 24 h post-injection. **C.** *In vivo* optical imaging and BLI of 4T1-fLuc tumor-bearing BALB/c mice 24 h after injection of GO(HPPH)-PEG-HK. **D, E.** Representative optical images of (D) and quantitative analysis of lung uptake (E) of GO(HPPH)-PEG-HK by 4T1-fLuc tumor-bearing and normal BALB/c mice at 24 h post-injection. *, P <0.05; **, P <0.01. Adapted with permission from 123, copyright 2017 Amer Chemical Soc. GO: graphene oxide; HPPH: 2-[1-hexyloxyethyl]-2-devinyl pyropheophorbide-alpha; HK: tumor integrin αvβ6-targeting peptide; PEG: polyethylene glycol.

Yu *et al.* 123 synthesized graphene oxide nanoprobes loaded with the PS photochlor (HPPH) and conjugated to integrin αvβ6-trageting peptides for tumor-specific PDT. The probe was also labeled with radionuclide and optical dyes, which enabled non-invasive monitoring of in-situ and distal tumor infiltration by CD8^+^ T cells after PDT by single photon emission computed tomography (SPECT) and NIR FI (**Figure 8**), thus confirming the key role of CD8^+^ T cells in PDT-mediated immunotherapy. In addition, the nanoprobes effectively inhibited tumor metastasis by direct killing of tumor cells *in situ* and turning them into “autologous vaccines” that can activate the anti-tumor immune response and generate long-term immune memory.

However, PDT alone can achieve very limited activation of innate and adaptive immune responses, which is inadequate for distant tumors. Immunoadjuvants such as CpG can enhance photodynamic immunotherapy by inducing DC activation and promoting the secretion of pro-inflammatory IL12p70 and TNF-α 123. Zhi *et al.* 124 synthesized NIR/ROS-responsive black phosphorus quantum dots (BPQD) vesicles (BPNVs) by self-assembly of polyethylene glycol and ROS-sensitive poly (propylene sulfide) (PPS), and encapsulated CpG in those vesicles (Bpnvs CpG). Following accumulation of BPNVs at the tumor site, they were irradiated by NIR laser to produce high levels of ROS that triggered transformation of hydrophobic PPS to hydrophilic polymer. This in turn dissociated CpG from BPNVs, which then penetrated into the deeper tumor tissues and was captured by the APCs along with tumor antigens, resulting in cytokine secretion and anti-tumor immune response. In addition, the dissociation and release of CpG was controlled by PAI. Thus, BPNVs-CPG achieved effective photodynamic immunotherapy *in vivo*, which inhibited the proliferation of tumor cells and blocked distant tumor growth and metastasis.

**Figure 9:**
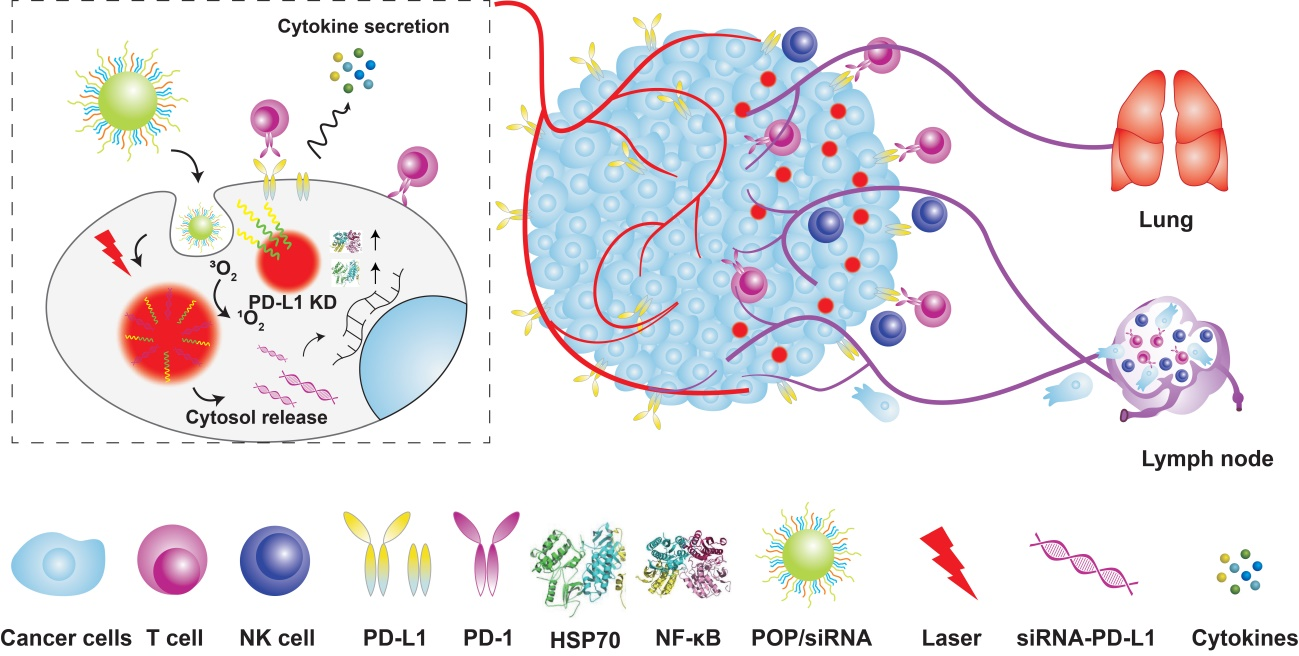
** Schematic illustration of the acid-responsive micelleplexes for PD-L1 blockade-enhanced photodynamic cancer immunotherapy.** Adapted with permission from 126, copyright 2016 Amer Chemical Soc. HSP70: heat shock protein 70; KD: knockdown; NK cell: natural killer cell; NF-κB: nuclear factor kappa B; PD-L1: programmed cell death 1 ligand; PD-1: programmed cell death receptor 1; siRNA: small interference RNA.

Tumor cells suppress T cell activity through PD-L1 and PD-1 immune checkpoints, which can neutralize PDT-mediated tumor immunotherapy. ICB can reverse the negative regulatory signals between immune cells and tumor cells, and increase the anti-tumor immune response of PDT 125. Dang *et al.* 126 designed multifunctional nanoprobes for photodynamic immunotherapy by integrating pH-responsive PDPA micelles, pheophyllin A (PPa) PS and small interference RNA (siRNA) to block PD-1/PD-L1 interaction (**Figure 9**). The homo fluorescence resonance energy transfer (homo-FRET) between PPa molecules quenched the fluorescence of the micelles. The acidic TME protonated the tertiary amine of PDPA and released PPa, which emitted fluorescence signals for tumor imaging. In addition, laser irradiation of the PS induced ROS formation, and photodynamic tumor ablation released antigens that were presented by the APCs to stimulate adaptive anti-tumor immune response. At the same time, the PD-L1-specific siRNA reversed immunosuppression by silencing the expression of PD-L1 on tumor cells. Compared to PDT alone, the combination of PDT and PD-L1 blockade significantly inhibited tumor growth and distant metastasis in a B16-F10 melanoma model.

Indoleamine 2,3-dioxygenase (IDO) is the rate limiting enzyme of the tryptophan metabolic pathway, and beaks down tryptophan to kynurenine. Increased tryptophan consumption and kynurenine accumulation promotes immune escape of tumor cells 127. Therefore, IDO is a key target of tumor immunotherapy 128. Ang *et al.* 129 showed that inactivating IDO-1 overcame PDT-induced adaptive immune resistance. They combined PEG-PS with NLG919, a prodrug of IDO-1 inhibitors, to synthesize a prodrug vesicle that specifically accumulated at the tumor site. Following cleavage of the PEG corona by matrix metalloproteinase-2 (MMP-2), the PS penetrated into the deep layers of the tumor for FI and PDT. Compared to PDT alone, photodynamic immunotherapy mediated by prodrug vesicles enhanced the immune response against CT26 and 4T1 tumor models and significantly inhibit tumor recurrence, especially for the IDO-overexpressing CT26 tumors. This differential tumor-specific therapeutic potential highlights the importance of IDO-1 expression for efficient photodynamic immunotherapy, and provides novel insights into the development of new multifunctional nanoprobes to overcome adaptive immune resistance.

## Image-guided multi-mode therapy combined with immunotherapy

As discussed in previous sections, chemo-immunotherapy, photothermal immunotherapy and photodynamic immunotherapy can achieve synergistic (1+1>2) therapeutic effects compared to any monotherapy. In addition, the combination of three or more modalities can further improve the therapeutic effect on tumors 130 by compensating for the disadvantages of each monotherapy 131,132. Integrating three or more different therapeutic methods into a single nanoprobe could have a better therapeutic effect than the single or dual-mode treatment 133,134 at lower doses, thereby avoiding the side effects of high drug dosage. Multiple therapeutic and imaging compounds can be assembled into a nanostructure through physical adsorption and chemical forces for multi-mode image-guided multi-mode therapy.

PTT and PDT are noninvasive and highly selective 135, and rely on photoactivation to convert light to local hyperthermia 136 or ROS 120 respectively and trigger tumor cell apoptosis. Several nanoprobes have been developed for combination PDT/PTT in recent years 137, especially the carbon-based nanoprobes that have the advantages of simple manufacture, strong NIR absorption and high photothermal conversion efficiency 138. Hong *et al.* 139 designed biodegradable carbon-silica multifunctional nanoprobes (CSN) with immunoadjuvant and PAI abilities for image-guided PDT/PTT. The nanoprobes effectively inhibited the growth of 4T1 and patient-derived xenograft (PDX) tumor models by 93.2% and 92.5% respectively. In addition, CSN was degraded into particles smaller than 5.5 nm upon NIR irradiation, and were excreted *via* the urine given the kidney filtration threshold (KFT) of ~5.5 nm, thus avoiding the long-term toxicity of carbon-based nanoprobes.

**Figure 10:**
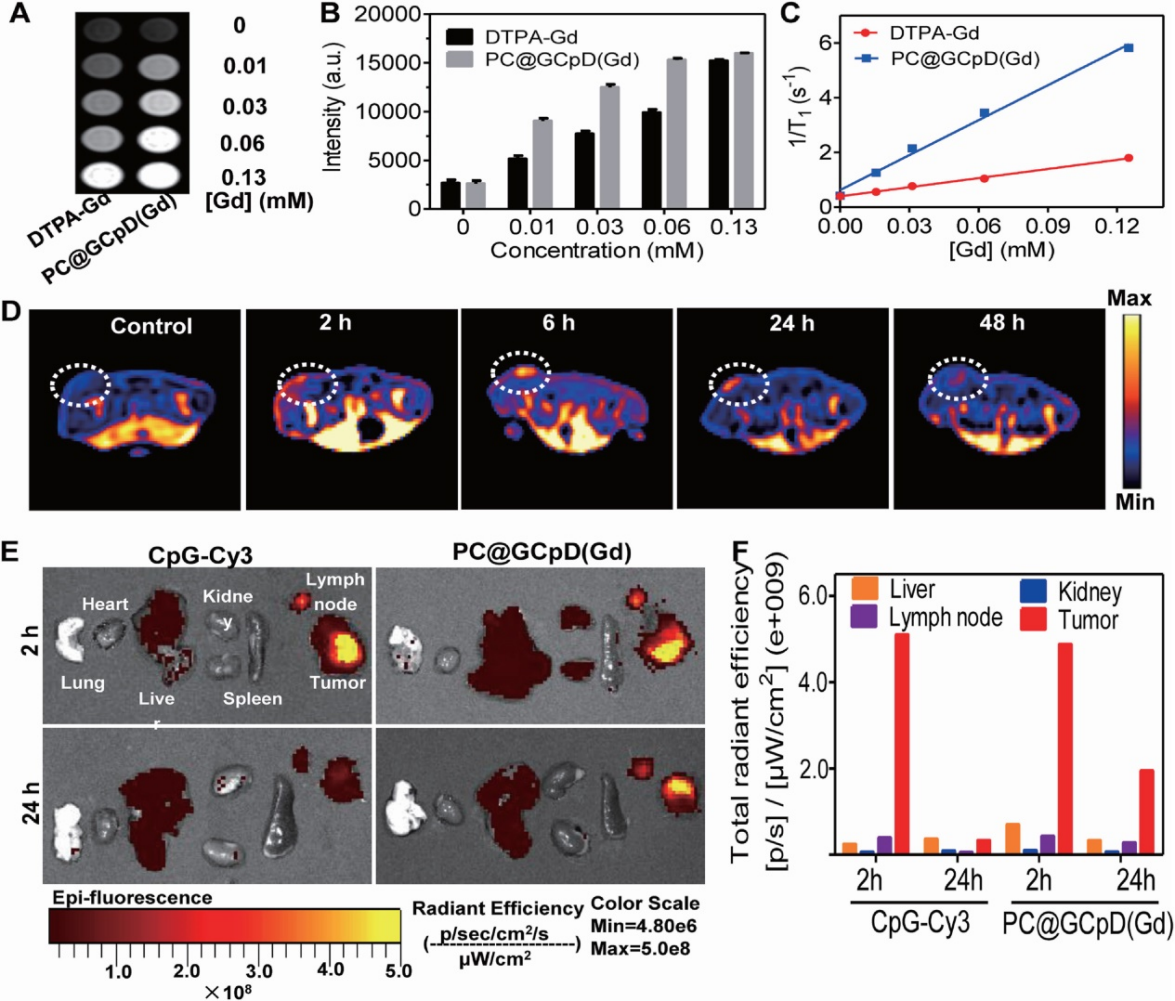
***In vivo* T_1_-weighted MRI and BioFI of PC@GCpD(Gd). A.** T1-weighted MRI signal of PC@GCpD(Gd) and DTPA-Gd measured at different gadolinium concentrations. **B.** Intensity comparison of PC@GCpD(Gd) and DTPA-Gd measured at different gadolinium concentrations. **C.** Relaxation rate of PC@GCpD(Gd) and DTPA-Gd measured at different gadolinium concentrations. **D.** T1-weighted MRI of EMT6 tumor-bearing mice at different time points after PC@GCpD(Gd) administration; white circles highlight the tumor site. **E.** The FI images of tumors and main tissues from mice treated with PC@GCpD(Gd) and free CpG-Cy3 for 2 h and 24 h. **F.** Quantitative comparison of tumors and main tissues from mice treated with PC@GCpD(Gd) and free CpG-Cy3 for 2 h and 24 h. Adapted with permission from 140, copyright 2019 Elsevier Sci Ltd. DTPA: diethylenetriaminepentaacetic acid; CpG: cytosine-phosphate-guanine.

Chun *et al.* 140 conjugated graphene quantum dots (GQDs) with Ce6 and coated them with polydopamine to form a functional photosensitive GQD complex (GCpD), which was then integrated with CpG and Gd^3+^/Cy3 MRI/FI contrast agent (PC@GCpD(Gd)) for cancer photoimmunotherapy and dual-mode imaging. The probes effectively killed tumor cells through GCpD-mediated PTT and PDT. In addition, CpG activated the innate immunity-related Toll-like receptor 9 (TLR9), resulting in continuous secretion of pro-inflammatory cytokines, DC maturation, and T lymphocyte activation and infiltration. PC@GCPD(Gd) had a strong inhibitory effect on the EMT6 mouse breast tumors under laser irradiation, indicating synergistic photothermal, photodynamic and immunotherapy. Furthermore, dual-mode MRI/FI can track the* in vivo* biodistribution of PC@GCPD(Gd). The MRI signals in the tumor were strongest at 6 h after administration and persisted after 48 h, indicating that PC@GCPD(Gd) can rapidly accumulate in the tumor tissues and remain *in situ* for a longer time, thereby maximizing the therapeutic effects. The MRI findings were also confirmed by FI of the tumors and other organs (**Figure 10**). PC@GCPD(Gd) also migrated to the draining lymph nodes, which is the primary site of DC maturation and activation of antigen-specific T cells for immunotherapy.

Although chemotherapy is the mainstay of cancer treatment, it is fraught with disadvantages such as non-specific action, low bioavailability, inability to overcome tumor heterogeneity and development of multidrug resistance (MDR), resulting in serious systemic side effects and tumor recurrence 141. The integration of non-invasive photodynamic 142, photothermal 143 and photodynamic photothermal 144 with chemotherapy in a single nanosystem can greatly improve the latter's efficacy while minimizing adverse side effects. In addition, chemotherapy, PTT and PDT can induce ICD to stimulate the immune system, which when combined with ICB can target the metastatic cancer cells and generate immune memory.

**Figure 11:**
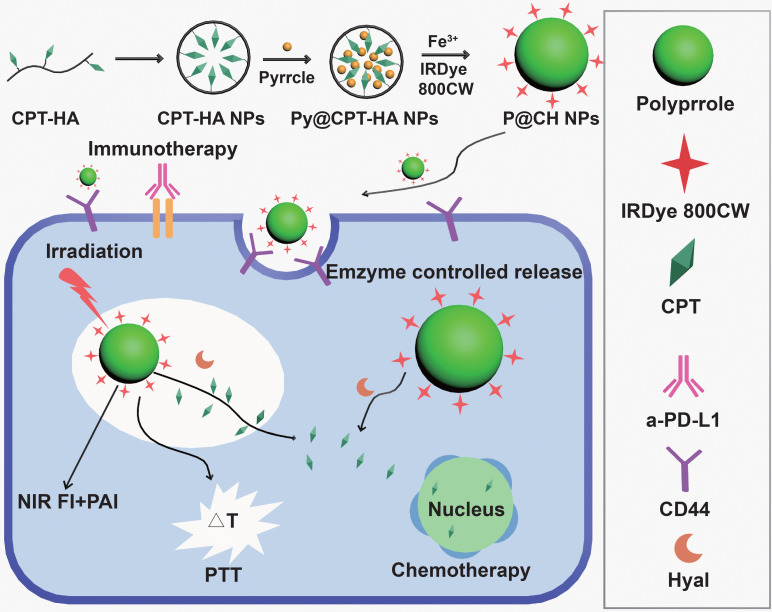
**Schematic illustration of the formation and functions of multifunctional nanoprobes.** Adapted with permission from 144, copyright 2019 Elsevier Sci Ltd. CPT: camptothecin; HA: hyaluronic acid; Hyal: hyaluronidase; NIR: the near infrared; FI: fluorescence imaging; PTT: photothermal therapy; PAI: photoacoustic imaging; a-PD-L1: atezolizumab.

Camptothecin can effectively kill tumor cells in the S phase and G2 phase, but its high toxicity limits clinical applications. Sun *et al.* 144 synthesized multifunctional nanoprobes by coupling CD44-targeted hyaluronic acid and camptothecin to encapsulate polypyrrole, and attached the NIR fluorescent dye IRDye800CW on the surface. The multifunctional nanoprobes (**Figure 11**) combined the chemotherapy function of camptothecin, the photoacoustic and photothermal functions of polypyrrole, and NIR FI capacity of IRDye800CW. Breast tumor-bearing mice were injected with P@CH and laser irradiated, followed by five doses of anti-PD-L1 antibodies every three days. The nanoparticles not only mitigated the toxicity of camptothecin in normal tissues but also produced anti-tumor immune response *via* PTT or chemotherapy-mediated cell death 145, eventually clearing the primary tumor cells and preventing tumor recurrence and metastasis in combination with immunotherapy 146,147.

**Figure 12:**
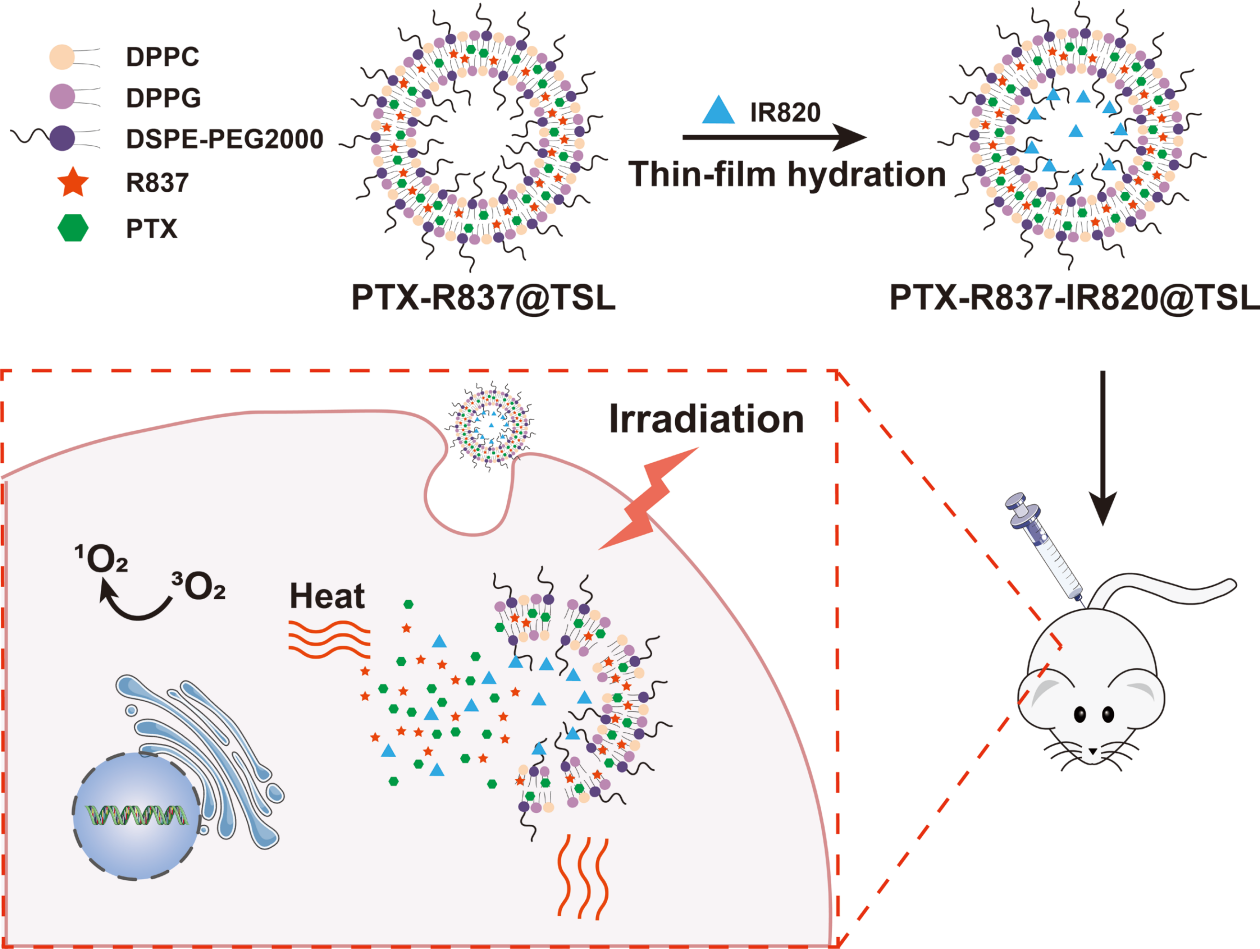
** Schematic illustration of the synthesis of PTX-R837-IR820@TSLcomplexes and the mechanism of combining NIR-mediated PDT /PTT with chemotherapy for cancer therapy.** Adapted with permission from 133, copyright 2019 Wiley-V C H Verlag Gmbh. DPPG: 1,2-dipaimitoylsn-glycerol-3-phospho-(1′-rac-glcerol); DSPE-PEG 2000: 1,2distearoyl-an-glycero-3-phosphoethanolamine-N [methoxy (polyethylene glycol)-2000]; DPPC: 1,2-dihexadecanoyl-snglycero-3-phosphocholine; IR820: a photosensitizer; PTX: paclitaxel; R837: imiquimod; TSL: thermosensitive liposomal.

Paclitaxel (PTX) is a commonly used chemotherapeutic drug 148,149 with good anticancer activity but low water solubility, which limits its applications. Therefore, a PTX loading system that can reduce its side effects and the risk of drug resistance would be of clinical significance. Xian *et al.* 133 encapsulated PTX, PS (IR820) and the TLR7 agonist imiquimod (R837) in a thermosensitive liposome (TSL) (**Figure 12**). NIR irradiation of PTX-R837-IR820@TSL at 808 nm led to the conversion of light energy into thermal energy and singlet oxygen by IR820, thus triggering PTT and PDT. Hyperthermia not only killed the tumor cells directly but also ruptured the TSLs to release the encapsulated PTX for a chemotherapeutic effect. Furthermore, R837 also acted as an immune adjuvant that augmented the immune response to the antigens released from the ablated tumors by triggering DC maturation and secretion of cytokines 59. Thus, PTX-R837-IR820@TSL can deliver chemotherapy drugs to target sites and enhance their accumulation to maximize the therapeutic effect. Taken together, the combination of PTT/PDT with chemotherapy and immunotherapy can synergistically inhibit tumor growth and metastasis, and achieve a stronger therapeutic effect.

## Conclusion and outlook

Cancer immunotherapy has the advantages of strong specificity, excellent durability and high safety, and is often called the “green therapy” for cancer. However, the current immunotherapeutic strategies are still limited by tumor heterogeneity, complexity of the TME, off-target effects and low immunogenicity. It is clinically challenging to “awaken” the immune response in such a complex system. Multifunctional nanoprobes have heralded a new era of cancer immunotherapy that can overcome existing limitations.

Multifunctional nanoprobes can effectively and continuously deliver tumor antigens and immune adjuvants to DCs and other APCs, thereby increasing antigen presentation and resulting in a sustained immune response. Furthermore, incorporation of contrast agents in multifunctional nanoprobes can achieve image-guided precise cancer immunotherapy. Multi-mode imaging combines the advantages of two or more imaging methods, for example high spatial resolution and high sensitivity, which can significantly improve cancer diagnosis. In addition, molecular imaging can visualize the interactions between immune cells and tumor cells in the TME, such as tumor T cell infiltration, cancer cell killing and migration of myeloid cells, and shed light on the mechanisms underlying immunotherapy.

The efficacy of cancer immunotherapy can be enhanced when combined with other therapies that further strengthen host immunity and overcome the immunosuppressive TME. For example, chemotherapy, PDT and PTT trigger the release of tumor antigens from the dying cells, which elicits a systemic anti-tumor immune response. When combined with ICB, the immune cells can be activated to attack metastatic cancer cells and generate long-term immune memory to inhibit tumor recurrence. Multifunctional nanoprobes that combine different monotherapies with immunotherapy, as well as multiple imaging modalities, can significantly improve the therapeutic effect against residual tumor cells.

Despite the encouraging results with pre-clinical models, there are several hurdles in the clinical applications of multifunctional nanoprobes. It is crucial to optimize the dose of drugs loaded into nanoprobes in order to minimize toxicity and maximize the synergistic effects. Although nanoprobes can accumulate in tumor tissues through enhanced permeability and retention effect (EPR), they are easily intercepted by the mononuclear phagocytic system (MPS) before reaching the tumor site, which not only decreases the effective dosage but also leads to off-target effects. While prolonging blood nanoprobes circulation can increase their accumulation at the tumor site, it can also increase their exposure to the immune system, generating a more complex immune response. The TME is physiologically complex, and differs from normal tissues in terms of uneven blood flow, hypoxia and acidic pH value among others. Therefore, nanoprobes responsive to hypoxia, acidity, redox, and tumor-specific enzymes, nucleic acids etc. can release their cargo in the TME in a controlled manner, with minimal effects on healthy tissues. Nevertheless, it is challenging to cross the immune defense barrier and achieve accurate and efficient drug delivery. One major concern is that animal models and cell lines cannot simulate the complexity and heterogeneity of human tumors and the TME, which is responsible for high disparity between patients in their response to immunotherapies. In addition, subcutaneous xenografts cannot replicate the immune status of tumors *in situ*. Finally, some immunotherapies may have significant side effects in humans that animal models cannot predict.

While multifunctional nanoprobes are a step towards safe and effective immunotherapy, and may even replace the current therapeutic strategies, a deeper understanding of the complex interactions between cancer cells and the immune system is needed to increase therapeutic efficacy.

## Figures and Tables

**Table 1:** Image-guided tumor immunotherapy using multifunctional nanoprobes

Immunotherapeutic agents	Delivery platform or modality	Imaging type	Model/Cell type	Refs
Anti-EpCAM/anti-CD3 encoding MC.DNA	PEI-C7-SPIO nanomicelles	MRI	HEK293T/HUVEC/MCF-10A/C17.2 NSCs	40
T cells	Au NPs	CT/FI	Mouse melanoma	41
Licor800 + PD-L1-mAb	Licor800 + PD-L1-mAb	SPECT/CT	Mouse breast cancer	42
Anti-PD-L1-mAb	ErNPs + PbS QDs	FI	Mouse colorectal cancer	43
IRDye800CW + Gd-DOTA + anti-PD-L1	Cerasome NPs	FI/MRI	Mouse breast cancer; mouse colorectal cancer	44
Aza-BODIPY + Gd-chelating MR probe	Aza-BODIPY + Gd-chelating; MR probe	FI/MRI	RAW 264.7 cells/BMDCs	45
InGaP/ZnS QDs	PFD nanodroplets	FI/MRI	NK92MI	46
anti-PD-L1	snowflake-like Au nanocarriers	CT	Mouse Prostate Cancer	47
CPG	Au NPs	CT	Mouse melanoma	48

**Table 2:** Multifunctional nanoprobes combined with immunotherapy guided by medical imaging

Immunotherapeutic agents	Delivery platform or modality	Imaging type		Model	Refs
**Chemoimmunotherapy**					
NLG 919 + OXA	Photosensitive NPs	FI		Mouse breast cancer	82
DOX + Cy7	Melittin-RADA32 hybrid peptide hydrogel	FI		Mouse melanoma	92
DOX + CPG	CpG self-crosslinking NPs	Dual-FI		Mouse melanoma	93
SN_38_ + BLZ-945	Au NPs	PAI		Mouse breast cancer	90
DOX + SDF-1α	Ag2Se QDs	FI		Mouse breast cancer	150
Paclitaxel + IRDye800CW + PD-L1-PCI-Gd	Nanohybrid liposomal cerasome NPs	FI/MRI		Mouse breast cancer; mouse colon tumor	44
**Photothermal immunotherapy**					
CpG self-crosslinked NPs	IR820-conjugated hydrogel	Dual-FI		Mouse melanoma	108
CPG	SPIO	PAI/MRI		Mouse breast cancer	112
Anti-PD-L1-peptide	Au@Pt NPs	PAI		Mouse breast cancer	136
Resiquimod + CDs	Polydopamine	FI/MSOT		Mouse breast cancer	151
Human CIK cells	Au NR@SiO_2_	PAI		Mouse gastric cancer	152
FNPs/rGO-PEG NPs	FNPs/rGO-PEG NPs	MRI		Mouse breast cancer	153
anti-PD-L1 peptides	Au@Pt	PAI		Mouse breast cancer	136
R837	MoS_2_-CuO heteronanocomposites	CTI/IRT/MRI		Mouse colon cancer	154
**Photodynamic immunotherapy**					
HPPH	Graphene oxide	SPECT/CT		Mouse breast cancer	123
CPG	BPQD vesicles	PAI		Mouse breast cancer	124
siRNA	Positively charged hybrid micelles,	FI		Mouse melanoma	126
NLG 919	A tumor-microenvironment-sheddable prodrug vesicle	FI		Mouse colorectal cancer; mouse breast cancer	129
Anti-PD-L1 Ab + IRDye800CW + DOTA-Gd + porphyrin	Cerasome NPs	FI/MRI		Mouse colorectal cancer	155
CPG + Ce6	Mesoporous silica NPs	PET		Mouse colorectal cancer; mouse melanoma	120
Cu_2_MoS_4_/Au heterostructures	Cu_2_MoS_4_/Au heterostructures	PAI/CT		Mouse cervical cancer	156
**Photodynamic-photothermal-immunotherapy**					
Carbon-silica nanocomposite	Carbon-silica nanocomposite	PAI		Mouse breast cancer; patient-derived xenograft model	139
Ce6 + CPG + Gd^3+^/Cy3	Graphene QDs	MRI/FI		Mouse breast cancer	140
Black phosphorus	Black phosphorus	PAI/FI		Mouse breast cancer	157
CPG + aPD-L1	Cu_9_S_5_	IRT		Mouse breast cancer	158
**Photothermal-chemotherapy-immunotherapy**					
IRDye800CW + polypyrrole	CPT-conjugated HA shell	PAI/FI		Mouse breast cancer	134
DOX + anti-PD-1	Ag@PDA	PAI/CT/IRT		Mouse breast cancer	159
anti-PD-1	CuS	PAI/USI		Mouse breast cancer	160
**Photodynamic-photothermal-chemotherapy-immunotherapy**					
PTX + R837 + IR820	Thermosensitive liposomal	FI	Mouse gastric carcinoma		133
ICG + PTX + CAT	Au nanoshells	IRT	Mouse cervical cancer		130
					

## References

[1] Bray F, Ferlay J, Soerjomataram I, Siegel RL, Torre LA, Jemal A (2018). Global cancer statistics 2018: GLOBOCAN estimates of incidence and mortality worldwide for 36 cancers in 185 countries. CA Cancer J Clin.

[2] Weissleder R, Pittet MJ (2008). Imaging in the era of molecular oncology. Nature.

[3] Simon R, Wang SJ (2006). Use of genomic signatures in therapeutics development in oncology and other diseases. Pharmacogenomics J.

[4] Cha R, Li J, Liu Y, Zhang Y, Xie Q, Zhang M (2017). Fe_3_O_4_ nanoparticles modified by CD-containing star polymer for MRI and drug delivery. Colloid Surfaces B.

[5] Wang Y, Xiong Z, He Y, Zhou B, Qu J, Shen M (2018). Optimization of the composition and dosage of PEGylated polyethylenimine-entrapped gold nanoparticles for blood pool, tumor, and lymph node CT imaging. Mat Sci Eng C-Mater.

[6] Yang T, Tang Y, Liu L, Lv X, Wang Q, Ke H (2017). Size-Dependent Ag_2_S nanodots for second near-infrared fluorescence/photoacoustics imaging and simultaneous photothermal therapy. ACS Nano.

[7] Yu WJ, Yu N, Wang ZJ, Li X, Song C, Jiang RQ (2019). Chitosan-mediated green synthesis and folic-acid modification of CuS quantum dots for photoacoustic imaging guided photothermal therapy of tumor. J. Colloid Interface Sci.

[8] Lei Z, Ding L, Yao C, Mo F, Li C, Huang Y (2019). A highly efficient tumor-targeting nanoprobe with a novel cell membrane permeability mechanism. Adv Mater.

[9] Zhang C, Xie H, Zhan T, Zhang J, Chen B, Qian Z (2019). A new mitochondrion targetable fluorescent probe for carbon monoxide-specific detection and live cell imaging. Chem Commun.

[10] Zhou H, Tang J, Zhang J, Chen B, Kan J, Zhang W (2019). A red lysosome-targeted fluorescent probe for carboxylesterase detection and bioimaging. J Mater Chem B.

[11] Xuan Y, Yang XQ, Song ZY, Zhang RY, Zhao DH, Hou XL (2019). High-Security multifunctional nano-bismuth-sphere-cluster prepared from oral gastric drug for CT/PA dual-mode imaging and chemo-photothermal combined therapy *in vivo*. Adv Funct Mater.

[12] Taube JM, Klein A, Brahmer JR, Xu H, Pan X, Kim JH (2014). Association of PD-1, PD-1 ligands, and other features of the tumor immune microenvironment with response to anti-PD-1 therapy. Clin Cancer Res.

[13] Lin WW, Karin M (2007). A cytokine-mediated link between innate immunity, inflammation, and cancer. J Clin Invest.

[14] Hahn AW, Gill DM, Pal SK, Agarwal N (2017). The future of immune checkpoint cancer therapy after PD-1 and CTLA-4. Immunotherapy.

[15] Shi GN, Zhang CN, Xu R, Niu JF, Song HJ, Zhang XY (2017). Enhanced antitumor immunity by targeting dendritic cells with tumor cell lysate-loaded chitosan nanoparticles vaccine. Biomaterials.

[16] Zhu G, Zhang F, Ni Q, Niu G, Chen X (2017). Efficient nanovaccine delivery in cancer immunotherapy. ACS Nano.

[17] Jin H, Qian Y, Dai Y, Qiao S, Huang C, Lu L (2016). Magnetic enrichment of dendritic cell vaccine in lymph node with fluorescent-magnetic nanoparticles enhanced cancer immunotherapy. Theranostics.

[18] Marshall JS, Warrington R, Watson W, Kim HL (2018). An introduction to immunology and immunopathology. Allergy Asthma Cl Im.

[19] Li J, Cha R, Zhang Y, Guo H, Long K, Gao P (2018). Iron oxide nanoparticles for targeted imaging of liver tumors with ultralow hepatotoxicity. J Mater Chem B.

[20] Sun Z, Huang G, Ma Z (2020). Synthesis of theranostic anti-EGFR ligand conjugate iron oxide nanoparticles for magnetic resonance imaging for treatment of liver cancer. J Drug Deliv Sci and Tec.

[21] Hao Y, Zhou X, Li R, Song Z, Min Y (2020). Advances of functional nanomaterials for cancer immunotherapeutic applications. WIREs Nanomed Nanobiotechnol.

[22] Jafari S, Lavasanifar A, Hejazi MS, Maleki-Dizaji N, Mesgari M, Molavi O (2020). STAT3 inhibitory stattic enhances immunogenic cell death induced by chemotherapy in cancer cells. Daru.

[23] Liu H, Yang Q, Guo W, Lin H, Zhang F, Zhao J (2020). CoWO_4-x_-based nanoplatform for multimode imaging and enhanced photothermal/photodynamic therapy. Chem Eng J.

[24] Song Y, Wang Y, Wang S, Cheng Y, Lu Q, Yang L (2019). Immune-adjuvant loaded Bi_2_Se_3_ nanocage for photothermal-improved PD-L1 checkpoint blockade immune-tumor metastasis therapy. Nano Res.

[25] Wang R, He Z, Cai P, Zhao Y, Gao L, Yang W (2019). Surface-Functionalized modified copper sulfide nanoparticles enhance checkpoint blockade tumor immunotherapy by photothermal therapy and antigencapturing. ACS Appl Mater Interfaces.

[26] Schwartz LH, Litiere S, de Vries E, Ford R, Gwyther S, Mandrekar S (2016). RECIST 1.1-update and clarification: From the RECIST committee. Eur J Cancer.

[27] Victoria LC, Mauricio B (2015). Pseudoprogression and immune-related response in solid tumors. J Clin Oncol.

[28] Hochmair MJ, Schwab S, Burghuber OC, Krenbek D, Prosch H (2017). Symptomatic pseudo-progression followed by significant treatment response in two lung cancer patients treated with immunotherapy. Lung Cancer.

[29] Nam J, Son S, Park KS, Zou W, Shea LD, Moon JJ (2019). Cancer nanomedicine for combination cancer immunotherapy. Nat Rev Mater.

[30] Zhao Z, Zheng L, Chen W, Weng W, Song J, Ji J (2019). Delivery strategies of cancer immunotherapy: recent advances and future perspectives. J Hematol Oncol.

[31] Ou YC, Wen X, Bardhan R (2020). Cancer immunoimaging with smart nanoparticles. Trends Biotechnol.

[32] Saeed M, Xu Z, De Geest BG, Xu H, Yu H (2020). Molecular imaging for cancer immunotherapy: Seeing is believing. Bioconjugate Chem.

[33] Zheng G, Dai Z (2020). Molecular imaging. Bioconjugate Chem.

[34] Wei W, Jiang D, Ehlerding EB, Barnhart TE, Yang Y, Engle JW (2019). CD146-targeted multimodal image-guided photoimmunotherapy of melanoma. Adv Sci.

[35] Fu Q, Zhu R, Song J, Yang H, Chen X (2019). Photoacoustic imaging: Contrast agents and their biomedical applications. Adv Mater.

[36] Wahsner J, Gale EM, Rodriguez-Rodriguez A, Caravan P (2019). Chemistry of MRI contrast agents: Current challenges and new frontiers. Chem Rev.

[37] Chauhan R, El-Baz N, Keynton RS, James KT, Malik DA, Zhu M (2019). Targeted gold nanoparticle-oligonucleotide contrast agents in combination with a new local voxel-wise MRI analysis algorithm for *in vitro* imaging of triple-negative breast cancer. Nanomaterials.

[38] Lin J, Xin P, An L, Xu Y, Tao C, Tian Q (2019). Fe_3_O_4_-ZIF-8 assemblies as pH and glutathione responsive T-2-T-1 switching magnetic resonance imaging contrast agent for sensitive tumor imaging *in vivo*. Chem Commun.

[39] Hola K, Markova Z, Zoppellaro G, Tucek J, Zboril R (2015). Tailored functionalization of iron oxide nanoparticles for MRI, drug delivery, magnetic separation and immobilization of biosubstances. Biotechnol Adv.

[40] Cai J, Chen G, Jin R, Deng C, Huang S, Yuan X (2019). A core-shell polymeric-inorganic hybrid nanocomposite system for MRI-visible gene delivery application in cancer immunotherapy. J Ind Eng Chem.

[41] Rinat M, Katerina S, Oshra B, Menachem M, Miryam HF, Ronen Y (2015). Nanomedicine for cancer immunotherapy: Tracking cancer specific T-Cells *in vivo* with gold nanoparticles and CT imaging. ACS Nano.

[42] Samit C, Wojciech GL, Matthew G, Ala L, Bryan W, Polina SS (2016). A humanized antibody for imaging immune checkpoint ligand PD-L1 expression in tumors. Oncotarget.

[43] Zhong Y, Ma Z, Wang F, Wang X, Yang Y, Liu Y (2019). *In vivo* molecular imaging for immunotherapy using ultra-bright near-infrared-IIb rare-earth nanoparticles. Nat Biotechnol.

[44] Du Y, Liang X, Li Y, Sun T, Xue H, Jin Z (2018). Liposomal nanohybrid cerasomes targeted to PD-L1 enable dual-modality imaging and improve antitumor treatments. Cancer Lett.

[45] Kim EJ, Bhuniya S, Lee H, Kim HM, Shin WS, Kim JS (2016). *In vivo* tracking of phagocytic immune cells using a dual imaging probe with gadolinium-enhanced MRI and near-infrared fluorescence. ACS Appl Mater Interfaces.

[46] Lim YT, Cho MY, Kang JH, Noh YW, Cho JH, Hong KS (2010). Perfluorodecalin/[InGaP/ZnS quantum dots] nanoemulsions as ^19^F MR/optical imaging nanoprobes for the labeling of phagocytic and nonphagocytic immune cells. Biomaterials.

[47] Choi B, Choi H, Yu B, Kim DH (2020). Synergistic local combination of radiation and anti-programmed death ligand 1 immunotherapy using radiation-responsive splintery metallic nanocarriers. ACS Nano.

[48] Lee IH, Kwon HK, An S, Kim D, Kim S, Yu MK (2012). Imageable antigen-presenting gold nanoparticle vaccines for effective cancer immunotherapy *in vivo*. Angew Chem Int Edit.

[49] Wang P, Kim T, Harada M, Contag C, Huang X, Smith BR (2020). Nano-immunoimaging. Nanoscale Horiz.

[50] Cai JL, Chen GC, Jin RR, Deng CH, Huang SH, Yuan XX (2019). A core-shell polymeric-inorganic hybrid nanocomposite system for MRI-visible gene delivery application in cancer immunotherapy. J Ind Eng Chem.

[51] Zhi D, Zhang S, Qureshi F, Zhao Y, Cui S, Wang B (2013). Structure-activity relationship of carbamate-linked cationic lipids bearing hydroxyethyl headgroup for gene delivery. Colloids Surf B Biointerfaces.

[52] Bakenecker A, Ahlborg M, Debbeler C, Kaethner C, Lüdtke-Buzug K (2018). Magnetic particle imaging. Precision Medicine.

[53] Dong X, Yang A, Bai Y, Kong D, Lv F (2020). Dual fluorescence imaging-guided programmed delivery of doxorubicin and CpG nanoparticles to modulate tumor microenvironment for effective chemo-immunotherapy. Biomaterials.

[54] Xu J, Yu S, Wang X, Qian Y, Wu W, Zhang S (2019). High affinity of chlorin e6 to immunoglobulin G for lntraoperative fluorescence image-guided cancer photodynamic and checkpoint blockade therapy. ACS Nano.

[55] Meir R, Shamalov K, Betzer O, Motiei M, Horovitz-Fried M, Yehuda R (2015). Nanomedicine for cancer immunotherapy: Tracking cancer-specific T-Cells *in vivo* with gold nanoparticles and CT imaging. ACS Nano.

[56] Fay AP, Signoretti S, Callea M, Telo GH, McKay RR, Song J (2015). Programmed death ligand-1 expression in adrenocortical carcinoma: an exploratory biomarker study. J Immunother Cancer.

[57] Bedognetti D, Maccalli C, Al Bader SBJ, Marincola FM, Seliger B (2016). Checkpoint inhibitors and their application in breast cancer. Breast Care.

[58] Topalian SL, Taube JM, Anders RA, Pardoll DM (2016). Mechanism-driven biomarkers to guide immune checkpoint blockade in cancer therapy. Nat Rev Cancer.

[59] Xu J, Xu L, Wang C, Yang R, Zhuang Q, Han X (2017). Near-Infrared-Triggered photodynamic therapy with multitasking upconversion nanoparticles in combination with checkpoint blockade for immunotherapy of colorectal cancer. ACS Nano.

[60] Xing Y, Zhao J, Conti PS, Chen K (2014). Radiolabeled nanoparticles for multimodality tumor imaging. Theranostics.

[61] Natarajan A, Mayer AT, Reeves RE, Nagamine CM, Gambhir SS (2017). Development of novel immune PET tracers to image human PD-1 checkpoint expression on tumor-infiltrating lymphocytes in a humanized mouse model. Mol Imaging Biol.

[62] Nedrow R, Josefsson A, Park S, Ranka S, Roy S, Sgouros G (2017). Imaging of programmed cell death ligand 1: Impact of protein concentration on distribution of anti-PD-L1 SPECT agents in an immunocompetent murine model of melanoma. J Nucl Med.

[63] Chatterjee S, Lesniak WG, Gabrielson M, Lisok A, Wharram B, Sysa-Shah P (2016). A humanized antibody for imaging immune checkpoint ligand PD-L1 expression in tumors. Oncotarget.

[64] Ge X, Fu Q, Su L, Li Z, Zhang W, Chen T (2020). Light-activated gold nanorod vesicles with NIR-II fluorescence and photoacoustic imaging performances for cancer theranostics. Theranostics.

[65] Zhang Q, Zhou H, Chen H, Zhang X, He S, Ma L (2019). Hierarchically nanostructured hybrid platform for tumor delineation and image-guided surgery *via* NIR-II fluorescence and PET bimodal imaging. Small.

[66] Ma Z, Zhang M, Yue J, Alcazar C, Zhong Y, Doyle TC (2018). Near-Infrared IIb fluorescence imaging of vascular regeneration with dynamic tissue perfusion measurement and high spatial resolution. Adv Funct Mater.

[67] Farhood B, Najafi M, Mortezaee K (2019). CD8^+^ cytotoxic T lymphocytes in cancer immunotherapy: A review. J Cell Physiol.

[68] Jiang P, Gu S, Pan D, Fu J, Sahu A, Hu X (2018). Signatures of T cell dysfunction and exclusion predict cancer immunotherapy response. Nat Med.

[69] Zhu S, Tian R, Antaris AL, Chen X, Dai H (2019). Near-Infrared-II molecular dyes for cancer imaging and surgery. Adv Mater.

[70] Chen B, Li C, Zhang J, Kan J, Jiang T, Zhou J (2019). Sensing and imaging of mitochondrial viscosity in living cells using a red fluorescent probe with a long lifetime. Chem Commun.

[71] Xuan Y, Song XL, Yang XQ, Zhang RY, Song ZY, Zhao DH (2019). Bismuth particles imbedded degradable nanohydrogel prepared by one-step method for tumor dual-mode imaging and chemo-photothermal combined therapy. Chem Eng J.

[72] Xuan Y, Zhang RY, Zhao DH, Zhang XS, An J, Cheng K (2019). Ultrafast synthesis of gold nanosphere cluster coated by graphene quantum dot for active targeting PA/CT imaging and near-infrared laser/pH-triggered chemo-photothermal synergistic tumor therapy. Chem Eng J.

[73] Chen M, Betzer O, Fan Y, Gao Y, Shen M, Sadan T (2020). Multifunctional dendrimer-entrapped gold nanoparticles for labeling and tracking T cells *Via* dual-modal Computed Tomography and Fluorescence Imaging. Biomacromolecules.

[74] Chiossone L, Dumas PY, Vienne M, Vivier E (2018). Natural killer cells and other innate lymphoid cells in cancer. Nat Rev Immunol.

[75] Guillerey C, Huntington ND, Smyth MJ (2016). Targeting natural killer cells in cancer immunotherapy. Nat Immunol.

[76] Charoentong P, Finotello F, Angelova M, Mayer C, Efremova M, Rieder D (2017). Pan-cancer immunogenomic analyses reveal genotype-immunophenotype relationships and predictors of response to checkpoint blockade. Cell Rep.

[77] Simoni Y, Becht E, Fehlings M, Loh CY, Koo SL, Teng KWW (2018). Bystander CD8^+^ T cells are abundant and phenotypically distinct in human tumour infiltrates. Nature.

[78] Tsoutsou PG, Zaman K, Luesma SM, Cagnon L, Kandalaft L, Vozenin MC (2018). Emerging opportunities of radiotherapy combined with immunotherapy in the era of breast cancer heterogeneity. Front Oncol.

[79] Zheng Q, Lin D, Lei L, Li X, Shi S (2017). Engineered non-viral Gene vectors for combination cancer therapy: A review. J Biomed Nanotechnol.

[80] Bains SJ, Abrahamsson H, Flatmark K, Dueland S, Hole KH, Seierstad T (2020). Immunogenic cell death by neoadjuvant oxaliplatin and radiation protects against metastatic failure in high-risk rectal cancer. Cancer Immunol Immun.

[81] Wang Q, Ju X, Wang J, Fan Y, Ren M, Zhang H (2018). Immunogenic cell death in anticancer chemotherapy and its impact on clinical studies. Cancer Lett.

[82] Feng B, Hou B, Xu Z, Saeed M, Yu H, Li Y (2019). Self-Amplified drug delivery with light-inducible nanocargoes to enhance cancer immunotherapy. Adv Mater.

[83] Li W, Yang J, Luo L, Jiang M, Qin B, Yin H (2019). Targeting photodynamic and photothermal therapy to the endoplasmic reticulum enhances immunogenic cancer cell death. Nat Commun.

[84] Yang W, Zhang F, Deng H, Lin L, Wang S, Kang F (2020). Smart nanovesicle-mediated immunogenic cell death through tumor microenvironment modulation for effective photodynamic immunotherapy. ACS Nano.

[85] Schirrmacher V (2019). From chemotherapy to biological therapy: A review of novel concepts to reduce the side effects of systemic cancer treatment (Review). Int J Oncol.

[86] Ariyan CE, Brady MS, Siegelbaum RH, Hu J, Bello DM, Rand J (2018). Robust antitumor responses result from local chemotherapy and CTLA-4 blockade. Cancer Immunol Res.

[87] Lim M, Xia Y, Bettegowda C, Weller M (2018). Current state of immunotherapy for glioblastoma. Nat Rev Clin Oncol.

[88] Chattopadhyay S, Liu YH, Fang ZS, Lin CL, Lin JC, Yao BY (2020). Synthetic immunogenic cell death mediated by intracellular delivery of STING agonist nanoshells enhances anticancer chemo-immunotherapy. Nano Lett.

[89] Yu Z, Guo J, Hu M, Gao Y, Huang L (2020). Icaritin exacerbates mitophagy and synergizes with doxorubicin to induce immunogenic cell death in hepatocellular carcinoma. ACS Nano.

[90] Zhu R, Su L, Dai J, Li ZW, Bai S, Li Q (2020). Biologically responsive plasmonic assemblies for second near-infrared window photoacoustic imaging-guided concurrent chemo-immunotherapy. ACS Nano.

[91] Zhang Y, Shen TT, Kirillov AM, Liu WS, Tang Y (2016). NIR light/H_2_O_2_-triggered nanocomposites for a highly efficient and selective synergistic photodynamic and photothermal therapy against hypoxic tumor cells. Chem Commun.

[92] Jin H, Wan C, Zou Z, Zhao G, Zhang L, Geng Y (2018). Tumor ablation and therapeutic immunity induction by an injectable peptide hydrogel. ACS Nano.

[93] Dong X, Yang A, Bai Y, Kong D, Lv F (2020). Dual fluorescence imaging-guided programmed delivery of doxorubicin and CpG nanoparticles to modulate tumor microenvironment for effective chemo-immunotherapy. Biomaterials.

[94] Ovais M, Guo M, Chen C (2019). Tailoring nanomaterials for targeting tumor-associated macrophages. Adv Mater.

[95] Pathria P, Louis TL, Varner JA (2019). Targeting tumor-associated macrophages in cancer. Trends Immunol.

[96] Shen S, Li HJ, Chen KG, Wang YC, Yang XZ, Lian ZX (2017). Spatial targeting of tumor-associated macrophages and tumor cells with a pH-sensitive cluster nanocarrier for cancer chemoimmunotherapy. Nano Lett.

[97] Wang Y, Luan Z, Zhao C, Bai C, Yang K (2020). Target delivery selective CSF-1R inhibitor to tumor-associated macrophages *via* erythrocyte-cancer cell hybrid membrane camouflaged pH-responsive copolymer micelle for cancer immunotherapy. Eur J Pharm Sci.

[98] Cheng K, Ding Y, Zhao Y, Ye S, Zhao X, Zhang Y (2018). Sequentially responsive therapeutic peptide assembling nanoparticles for dual-targeted cancer immunotherapy. Nano Lett.

[99] Feng B, Zhou F, Hou B, Wang D, Wang T, Fu Y (2018). Binary cooperative prodrug nanoparticles improve immunotherapy by synergistically modulating immune tumor microenvironment. Adv Mater.

[100] Liu Y, Chen XG, Yang PP, Qiao ZY, Wang H (2019). Tumor microenvironmental pH and enzyme dual responsive polymer-liposomes for synergistic treatment of cancer immuno-chemotherapy. Biomacromolecules.

[101] Shang T, Yu X, Han S, Yang B (2020). Nanomedicine-based tumor photothermal therapy synergized immunotherapy. Biomater Sci.

[102] Cheng Y, Chang Y, Feng Y, Jian H, Tang Z, Zhang H (2018). Deep-Level defect enhanced photothermal performance of bismuth sulfide-gold heterojunction nanorods for photothermal therapy of cancer guided by computed tomography imaging. Angew Chem Int Edit.

[103] Sun T, Dou JH, Liu S, Wang X, Zheng X, Wang Y (2018). Second near-infrared conjugated polymer nanoparticles for photoacoustic imaging and photothermal therapy. ACS Appl Mater Interfaces.

[104] Guo L, Yan DD, Yang D, Li Y, Wang X, Zalewski O (2014). Combinatorial photothermal and immuno cancer therapy using chitosan-coated hollow copper sulfide nanoparticles. ACS Nano.

[105] Ye X, Liang X, Chen Q, Miao Q, Chen X, Zhang X (2019). Surgical tumor-derived personalized photothermal vaccine formulation for cancer immunotherapy. ACS Nano.

[106] Ma Y, Zhang Y, Li X, Zhao Y, Li M, Jiang W (2019). Near-Infrared II phototherapy induces deep tissue immunogenic cell death and potentiates cancer immunotherapy. ACS Nano.

[107] Nguyen HT, Tran KK, Sun B, Shen H (2012). Activation of inflammasomes by tumor cell death mediated by gold nanoshells. Biomaterials.

[108] Dong X, Liang J, Yang A, Qian Z, Kong D, Lv F (2019). Fluorescence imaging guided CpG nanoparticles-loaded IR820-hydrogel for synergistic photothermal immunotherapy. Biomaterials.

[109] Ming J, Zhang J, Shi Y, Yang W, Li J, Sun D (2020). A trustworthy CpG nanoplatform for highly safe and efficient cancer photothermal combined immunotherapy. Nanoscale.

[110] Umeki Y, Saito M, Kusamori K, Tsujimura M, Nishimura M, Takahashi Y (2018). Combined encapsulation of a tumor antigen and immune cells using a self-assembling immunostimulatory DNA hydrogel to enhance antigen-specific tumor immunity. J Control Release.

[111] Ruiz-de-Angulo A, Zabaleta A, Gomez-Vallejo V, Llop J, Mareque-Rivas JC (2016). Microdosed lipid-coated ^67^Ga-magnetite enhances antigen-specific immunity by image tracked delivery of antigen and CpG to lymph nodes. ACS Nano.

[112] Guo Y, Ran Y, Wang Z, Cheng J, Cao Y, Yang C (2019). Magnetic-responsive and targeted cancer nanotheranostics by PA/MR bimodal imaging-guided photothermally triggered immunotherapy. Biomaterials.

[113] Li Y, He L, Dong H, Liu Y, Wang K, Li A (2018). Fever-Inspired immunotherapy based on photothermal CpG nanotherapeutics: The critical role of mild heat in regulating tumor microenvironment. Adv Sci.

[114] Huang L, Li Y, Du Y, Zhang Y, Wang X, Ding Y (2019). Mild photothermal therapy potentiates anti-PD-L1 treatment for immunologically cold tumors *via* an all-in-one and all-in-control strategy. Nat Commun.

[115] Liu B, Cao W, Qiao G, Yao S, Pan S, Wang L (2019). Effects of gold nanoprism-assisted human PD-L1 siRNA on both gene down-regulation and photothermal therapy on lung cancer. Acta Biomater.

[116] Janko C, Ratschker T, Nguyen K, Zschiesche L, Tietze R, Lyer S (2019). Functionalized superparamagnetic iron oxide nanoparticles (SPIONs) as platform for the targeted multimodal tumor therapy. Front Oncol.

[117] Cheng HW, Tsao HY, Chiang CS, Chen SY (2021). Advances in magnetic nanoparticle-mediated cancer immune-theranostics. Adv Healthc Mater.

[118] Lan M, Zhao S, Liu W, Lee C.-S, Zhang W, Wang P (2019). Photosensitizers for photodynamic therapy. Adv Healthc Mater.

[119] Hou X, Tao Y, Pang Y, Li X, Jiang G, Liu Y (2018). Nanoparticle-based photothermal and photodynamic immunotherapy for tumor treatment. Int J Cancer.

[120] Green DR, Ferguson T, Zitvogel L, Kroemer G (2009). Immunogenic and tolerogenic cell death. Nat Rev Immunol.

[121] Kepp O, Senovilla L, Vitale I, Vacchelli E, Adjemian S, Agostinis P (2014). Consensus guidelines for the detection of immunogenic cell death. Oncoimmunology.

[122] Xu C, Nam J, Hong H, Xu Y, Moon JJ (2019). Positron emission tomography-guided photodynamic therapy with biodegradable mesoporous silica nanoparticles for personalized cancer immunotherapy. ACS Nano.

[123] Yu X, Gao D, Gao L, Lai J, Zhang C, Zhao Y (2017). Inhibiting metastasis and preventing tumor relapse by triggering host immunity with tumor-targeted photodynamic therapy using photosensitizer-loaded functional nanographenes. ACS Nano.

[124] Li Z, Hu Y, Fu Q, Liu Y, Wang J, Song J (2019). NIR/ROS-Responsive black phosphorus QD vesicles as immunoadjuvant carrier for specific cancer photodynamic immunotherapy. Adv Funct Mater.

[125] Lan G, Ni K, Xu Z, Veroneau SS, Song Y, Lin W (2018). Nanoscale metal-organic framework overcomes hypoxia for photodynamic therapy primed cancer immunotherapy. J Am Chem Soc.

[126] Wang D, Wang T, Liu J, Yu H, Jiao S, Feng B (2016). Acid-Activatable versatile micelleplexes for PD-L1 blockade enhanced cancer photodynamic immunotherapy. Nano Lett.

[127] Hornyak L, Dobos N, Koncz G, Karanyi Z, Pall D, Szabo Z (2018). The role of indoleamine-2,3-dioxygenase in cancer development, diagnostics, and therapy. Front Immunol.

[128] Liu M, Wang X, Wang L, Ma X, Gong Z, Zhang S (2018). Targeting the IDO1 pathway in cancer: From bench to bedside. J Hematol Oncol.

[129] Gao A, Chen B, Gao J, Zhou F, Saeed M, Hou B (2019). Sheddable prodrug vesicles combating adaptive immune resistance for improved photodynamic immunotherapy of cancer. Nano Lett.

[130] He Y, Cong C, He Y, Hao Z, Li C, Wang S (2019). Tumor hypoxia relief overcomes multidrug resistance and immune inhibition for self-enhanced photodynamic therapy. Chem Eng J.

[131] Dendy MS, Ludwig JM, Stein SM, Kim HS (2019). Locoregional therapy, immunotherapy and the combination in hepatocellular carcinoma: Future directions. Liver Cancer.

[132] Liu B, Cao W, Cheng J, Fan S, Pan S, Wang L (2019). Human natural killer cells for targeting delivery of gold nanostars and bimodal imaging directed photothermal/photodynamic therapy and immunotherapy. Cancer Biol Med.

[133] Meng X, Wang K, Lv L, Zhao Y, Sun C, Ma L (2019). Photothermal/Photodynamic therapy with immune-adjuvant liposomal complexes for effective gastric cancer therapy. Part Part Syst Char.

[134] Sun W, Du Y, Liang X, Yu C, Fang J, Lu W (2019). Synergistic triple-combination therapy with hyaluronic acid-shelled PPy/CPT nanoparticles results in tumor regression and prevents tumor recurrence and metastasis in 4T1 breast cancer. Biomaterials.

[135] Yan S, Zeng X, Tang Y, Liu BF, Wang Y, Liu X (2019). Activating antitumor immunity and antimetastatic effect through polydopamine-encapsulated core-shell upconversion nanoparticles. Adv Mater.

[136] Yang Q, Peng J, Shi K, Xiao Y, Liu Q, Han R (2019). Rationally designed peptide-conjugated gold/platinum nanosystem with active tumor-targeting for enhancing tumor photothermal-immunotherapy. J Control Release.

[137] Yong Y, Zhou L, Gu Z, Yan L, Tian G, Zheng X (2014). WS_2_ nanosheet as a new photosensitizer carrier for combined photodynamic and photothermal therapy of cancer cells. Nanoscale.

[138] Zhang JJ, Cheng FF, Li JJ, Zhu JJ, Lu Y (2016). Fluorescent nanoprobes for sensing and imaging of metal ions: Recent advances and future perspectives. Nano Today.

[139] Wang H, Pan X, Wang X, Wang W, Huang Z, Gu K (2020). Degradable carbon-silica nanocomposite with immunoadjuvant property for dual-modality photothermal/photodynamic therapy. ACS Nano.

[140] Wu C, Guan X, Xu J, Zhang Y, Liu Q, Tian Y (2019). Highly efficient cascading synergy of cancer photo-immunotherapy enabled by engineered graphene quantum dots/photosensitizer/CpG oligonucleotides hybrid nanotheranostics. Biomaterials.

[141] Wang H, Gao Z, Liu X, Agarwal P, Zhao S, Conroy DW (2018). Targeted production of reactive oxygen species in mitochondria to overcome cancer drug resistance. Nature Commun.

[142] Yuan Y, Liu J, Liu B (2014). Conjugated-polyelectrolyte-based polyprodrug: Targeted and image-guided photodynamic and chemotherapy with on-demand drug release upon irradiation with a single light source. Angew Chem Int Edit.

[143] Tan L, Huang R, Li X, Liu S, Shen YM, Shao Z (2017). Chitosan-based core-shell nanomaterials for pH-triggered release of anticancer drug and near-infrared bioimaging. Carbohydr Polym.

[144] Sun W, Du Y, Liang X, Yu C, Fang J, Lu W (2019). Synergistic triple-combination therapy with hyaluronic acid-shelled PPy/CPT nanoparticles results in tumor regression and prevents tumor recurrence and metastasis in 4T1 breast cancer. Biomaterials.

[145] Chen YL, Chang MC, Cheng WF (2017). Metronomic chemotherapy and immunotherapy in cancer treatment. Cancer Lett.

[146] Duan X, Chan C, Guo N, Han W, Weichselbaum RR, Lin W (2016). Photodynamic therapy mediated by nontoxic core-shell nanoparticles synergizes with immune checkpoint blockade to elicit antitumor immunity and antimetastatic effect on breast cancer. J Am Chem Soc.

[147] Gao L, Zhang C, Gao D, Liu H, Yu X, Lai J (2016). Enhanced anti-tumor efficacy through a combination of integrin alpha v beta 6-targeted photodynamic therapy and immune checkpoint inhibition. Theranostics.

[148] Reck M, Bondarenko I, Luft A, Serwatowski P, Barlesi F, Chacko R (2013). Ipilimumab in combination with paclitaxel and carboplatin as first-line therapy in extensive-disease-small-cell lung cancer: results from a randomized, double-blind, multicenter phase 2 trial. Ann Oncol.

[149] Wilke H, Muro K, Van Cutsem E, Oh SC, Bodoky G, Shimada Y (2014). Ramucirumab plus paclitaxel versus placebo plus paclitaxel in patients with previously treated advanced gastric or gastro-oesophageal junction adenocarcinoma (RAINBOW): a double-blind, randomised phase 3 trial. Lancet Oncol.

[150] Hao X, Li C, Zhang Y, Wang H, Chen G, Wang M (2018). Programmable chemotherapy and immunotherapy against breast cancer guided by multiplexed fluorescence imaging in the second near-infrared window. Adv Mater.

[151] Lu Q, Qi S, Li P, Yang L, Yang S, Wang Y (2019). Photothermally activatable PDA immune nanomedicine combined with PD-L1 checkpoint blockade for antimetastatic cancer photoimmunotherapy. J Mater Chem B.

[152] Yang Y, Zhang J, Xia F, Zhang C, Qian Q, Zhi X (2016). Human CIK cells loaded with Au nanorods as a theranostic platform for targeted photoacoustic imaging and enhanced immunotherapy and photothermal therapy. Nanoscale Res Lett.

[153] Wang L, Wang M, Zhou B, Zhou F, Murray C, Towner RA (2019). PEGylated reduced-graphene oxide hybridized with Fe_3_O_4_ nanoparticles for cancer photothermal-immunotherapy. J Mater Chem B.

[154] Jiang F, Ding B, Liang S, Zhao Y, Cheng Z, Xing B (2021). Intelligent MoS_2_-CuO heterostructures with multiplexed imaging and remarkably enhanced antitumor efficacy *via* synergetic photothermal therapy/chemodynamic therapy/immunotherapy. Biomaterials.

[155] Li Y, Du Y, Liang X, Sun T, Xue H, Tian J (2018). EGFR-targeted liposomal nanohybrid cerasomes: theranostic function and immune checkpoint inhibition in a mouse model of colorectal cancer. Nanoscale.

[156] Chang M, Hou Z, Wang M, Wang M, Dang P, Liu J (2020). Cu_2_MoS_4_/Au heterostructures with enhanced catalase-like activity and photoconversion efficiency for primary/metastatic tumors eradication by phototherapy-induced immunotherapy. Small.

[157] Zhang X, Tang J, Li C, Lu Y, Cheng L, Liu J (2021). A targeting black phosphorus nanoparticle based immune cells nano-regulator for photodynamic/photothermal and photo-immunotherapy. Bioact Mater.

[158] Zhou L, Chen L, Hu X, Lu Y, Liu W, Sun Y (2020). A Cu_9_S_5_ nanoparticle-based CpG delivery system for synergistic photothermal-, photodynamic- and immunotherapy. Commun Biol.

[159] Zeng X, Yan S, Di C, Lei M, Chen P, Du W (2020). “All-in-One” silver nanoprism platform for targeted tumor theranostics. ACS Appl Mater Interfaces.

[160] Zhang W, Zhang CC, Wang XY, Li L, Chen QQ, Liu WW (2020). Light-responsive core-shell nanoplatform for bimodal imaging-guided photothermal therapy-primed cancer immunotherapy. ACS Appl Mater Interfaces.

